# Fhl1, a new spatially specific protein, regulates vein graft neointimal hyperplasia

**DOI:** 10.1002/ctm2.70115

**Published:** 2024-12-05

**Authors:** Chaoqun Wang, Jiantao Chen, Zicong Feng, Bohao Jian, Suiqing Huang, Kangni Feng, Haoliang Liu, Zhuoming Zhou, Ziyin Ye, Jing Lu, Mengya Liang, Zhongkai Wu

**Affiliations:** ^1^ Department of Cardiac Surgery First Affiliated Hospital of Sun Yat‐Sen University Guangzhou China; ^2^ NHC Key Laboratory of Assisted Circulation Sun Yat‐Sen University Guangzhou China; ^3^ Department of Pathology First Affiliated Hospital of Sun Yat‐Sen University Guangzhou China; ^4^ School of Pharmaceutical Sciences Sun Yat‐Sen University Guangzhou China

**Keywords:** Fhl1, neointimal hyperplasia, spatial transcriptomics, vein graft remodelling

## Abstract

**Background:**

Vein grafts are commonly employed in revascularisation surgery for multivessel coronary artery disease, yet neointimal hyperplasia (NIH) remains a critical impediment to the long‐term patency of these grafts. Despite this, effective methods to precisely identify and target interventions for the neointima are still inadequate.

**Methods:**

In this study, Sprague–Dawley (SD) rats were used to establish an external jugular vein transplantation model, and the NIH pathophysiological process was tracked across 11 time points (0–35 days) using various histological stains. Spatial transcriptomics was performed on normal veins and 19‐day grafts to explore gene expression in neointimal regions. Immunohistochemical analysis identified neointima‐specific markers, while NIH progression was assessed in SD rats with four and a half LIM domains protein 1 (Fhl1) knockout and in human saphenous veins (HSV) with adenovirus‐mediated Fhl1 overexpression.

**Results:**

Typical neointimal formation commenced by day 11 postgrafting and peaked at day 19. Neointimal cells originated from newly generated α‐SMA(+) repair cells located outside the grafted vein, displaying a hybrid fibroblast‐smooth muscle cell phenotype. Spatial transcriptomics identified stable and sustained Fhl1 expression within the neointima throughout the entire NIH phase. Systemic knockout of Fhl1 in SD rats via the phosphoinositide 3‐kinase pathway exacerbated graft inflammation, heightened cell proliferation, and accelerated NIH. Conversely, FHL1 overexpression in cultured HSV suppressed NIH.

**Conclusion:**

These findings indicate that, following grafting into the arterial system, the newly formed repair cells external to the grafted vein play a pivotal role in NIH, with neointimal cells exhibiting stable and continuous Fhl1 expression. Fhl1 serves as a protective factor against NIH both in vivo and in HSV, likely due to its anti‐inflammatory and anti‐proliferative effects.

**Key points:**

- This study firstly used spatial transcriptomics technique to analyse the neointima and generated a specific neointimal transcriptomic atlas.
- Fhl1 exhibits specific and stable expression in the spatial region of the neointima. It has thus far the highest enrichment of expression in the neointima in NIH phases, suggesting that it is a prominent molecular biomarker of neointima.
- We generated rats with a Fhl1 deletion and found that insufficient Fhl1 expression caused an increase in the severity of vascular inflammation and proliferation during neointimal hyperplasia. Adenovirus‐mediated FHL1 overexpression in human saphenous vein have beneficial effects in preventing neointimal hyperplasia. These highlight its potential as a therapeutic target for mitigating vein graft failure associated with cardiovascular procedures.
- Spatial transcriptomics profiles and morphological observations demonstrated that a newly generated cell population outside the grafted vein with hybrid phenotype between SMCs and fibroblasts contributes to neointimal formation.

## Graphical abstract

1. Dominant cell type of neointima exhibited myofibroblast features and arose primarily from tissue external to the vein.
2. Among differentially expressed genes identified by spatial transcriptomics, Fhl1 was strongly expressed in neointima.
3. Fhl1 prevented NIH in vivo and cultured human veins through its anti‐inflammation and anti‐proliferation properties.

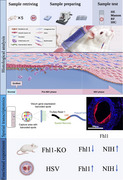

## 1 INTRODUCTION

Coronary artery disease (CAD), with myocardial infarction as its primary cardiovascular manifestation, remains a leading cause of morbidity and mortality globally.^1^ Coronary artery bypass grafting continues to be an effective revascularisation method, particularly for multivessel CAD.^2^ Despite the prevalent use of the internal mammary artery for left coronary revascularisation, the autologous saphenous vein remains the most frequently utilised conduit.^3^, ^4^ Vein grafts undergo vascular remodelling and neointimal hyperplasia (NIH), which significantly compromises long‐term patency.^5^, ^6^ Up to 60% of vein grafts fail within 10 years postoperatively, primarily due to NIH and atherogenesis.^7^, ^8^ To date, no intervention has been clinically validated to effectively prevent vein graft failure (VGF).^9^ Current postoperative treatments, such as statins and antiplatelet agents, offer only minimal efficacy in reducing NIH.^10^ While targeting NIH‐contributing cells shows therapeutic potential, reliable neointimal factors remain undiscovered.

In 1965, researchers at the University of Rochester first identified the neointima of vein grafts as white fibrous tissue.^11^ Vein grafts are dynamic, self‐remodelling tissues that adapt in response to haemodynamic stimuli and signals from the local microenvironment.^12^ The origin of the cells involved in NIH remains a subject of debate. While the majority of studies suggest that NIH predominantly arises from medial smooth muscle cells (SMCs),^13^, ^14^, ^15^, ^16^ alternative hypotheses propose an endothelial cell origin,^17^ and some suggest adventitial fibroblasts as the source.^18^ Most previous research only captured data from limited time points during NIH,^19^, ^20^ failing to dynamically monitor cell replacement during vascular remodelling. Furthermore, high‐throughput sequencing omics technologies have proven vital in investigating the mechanisms behind vein graft occlusion.^21^ Previous studies have used bulk RNA‐seq after dissociating vein grafts, yielding averaged data from all three spatial layers of the graft. However, research increasingly focuses on the spatial structure of the neointima, and traditional methods fail to preserve cell positioning within tissues, making it difficult to pinpoint markers specific to the neointima or identify key pathogenic genes.^22^ To address this spatial heterogeneity, numerous advanced methodologies have been developed.^23^, ^24^, ^25^ Spatial transcriptomics (ST), a next‐generation sequencing‐based technique, captures transcript information from across tissue regions while encoding spatial location data into the transcripts, making it ideal for investigating gene expression across different spatial regions of tissues.^26^, ^27^, ^28^, ^29^

This study aimed to investigate the dynamic morphological changes in vein graft remodelling and trace the transition of cell populations contributing to NIH. Additionally, ST was applied to uncover spatial gene expression profiles, enabling the identification of key molecular biomarkers and factors involved in NIH.

## 2 MATERIALS AND METHODS

### 2.1 Rat vein graft model

As previously described,^30^ all animal experiments were conducted with approval from the Institutional Animal Care and Use Committee, in accordance with the Guide for the Care and Use of Laboratory Animals of Sun Yat‐Sen University (Number SYSUIACUC2020‐000074). Procedures adhered to ARRIVE guidelines. Ten‐ to twelve‐week male SD (Sprague–Dawley) rats, weighing 250–350 g, underwent interposition bypass grafting (Figure S1). A single intraperitoneal injection of pentobarbital was conducted to anesthetise the SD rats (30 mg/kg, Sanofi, France). A segment of right external jugular vein from the SD rat was excised and preserved in low‐concentration heparin saline (5000 U/L, Sanofi, France). Systemic heparinisation was induced with an intravenous dose of 100 units of heparin. After isolating a section of the right common carotid artery, the two ends were clamped using microvascular clamps. Vein grafts were constructed with a cuff technique, utilising a plastic tube (Portex, .75 mm inner diameter, .94 mm outer diameter, Smiths Medical, UK). The arterial ends were everted over the cuff and secured with an 8‐0 silk ligature. The vein graft was inverted and inserted between the arterial ends, then fixed around the cuff with a second 8‐0 silk ligature. The incision was closed with 6‐0 sutures, and aspirin (20 mg/day, Pharmaceutical Formulations, Edison, NJ) was administered via oral gavage to prevent thrombus formation.

Patent grafts were harvested from five rats at each time point (control, 4 h, Day 1, Day 3, Day 7, Day 11, Day 14, Day 19, Day 21, Day 28, and Day 35) to monitor longitudinal morphological changes during NIH. Euthanasia was performed via a single intraperitoneal injection of an overdose of pentobarbital (300 mg/kg, Sanofi, France). Vein grafts were fixed in their perfused state to produce paraffin‐embedded specimens, ensuring cross‐sectional areas comparable to those observed in vivo. Grafts were immersed in 10% formalin for morphometric analysis.

### 2.2 Generation of Fhl1 knockout rats

Normal SD rats were used as wild‐type (WT) controls. The four and a half LIM domains protein 1 (Fhl1) gene (Gene ID: 25177), located on the X chromosome in rats, comprises seven exons, with the ATG start codon in exon 3 and the TAA stop codon in exon 7. Exons 3 through 7 were selected as the target region. The generation of Fhl1 knockout (KO) rats using the CRISPR/Cas9 system proceeded as follows: one‐cell stage zygotes were obtained by mating super‐ovulated SD females (Charles River, China) with SD males (Charles River, China). Superovulation was induced by injections of pregnant mare serum gonadotropin (PMSG; Prospec, Israel) and human chorionic gonadotropin (HCG; Prospec, Israel). Cas9 mRNA and guide RNA (gRNA) were synthesised through in vitro transcription (AM1354+AM1908, Thermo, USA). A solution containing .3 µM Cas9 mRNA and .75 µM gRNA was injected into the cytoplasm of pronuclear‐stage embryos. These embryos were then cultured overnight in KSOM medium (Millipore, Germany), and those that developed to the two‐cell stage were transferred into the oviducts of pseudo‐pregnant SD females (Charles River, China). The gRNA sequences were as follows:

gRNA‐A1: GCCAGACCTTATGCTGGCTAAGG;
gRNA‐A2: CTCAGTCGGGACGCACTGTGCGG;
gRNA‐B1: GCACTACTGCGCATGTACCTAGG;
gRNA‐B2: GCCCACTCTTGCCCTATGGATGG.

F0 founder animals were identified through PCR and subsequent sequence analysis, then bred with WT rats to assess germline transmission and produce F1 offspring. F2 homozygotes were obtained from the F1 generation. Genomic DNA was extracted from rat tail tips, and genotyping primers were designed as follows:

Primers1:

F1: 5′‐TGGCAGGAAGGCTTCTGGCTT‐3′;
R1: 5′‐GCCACATAGGGGAGTCAAACATTA‐3′ (Product size: 488 bp).

Primers2:

F1: 5′‐TGGCAGGAAGGCTTCTGGCTT‐3′;
R2: 5′‐CAGCTACAACAGTTACCTTAGCATC‐3′ (Product size: 778 bp).

The Fhl1 KO product length was 488 bp, while the WT product was 778 bp.

No significant differences were observed between Fhl1 KO and WT rats regarding physical appearance, behaviour, heart rate, or postoperative survival rates.

### 2.3 Histological and morphometric analysis

As previously described,^30^ serial paraffin‐embedded sections (3 µm) were prepared from tissue located .5 mm distal to the proximal cuff anastomosis. Haematoxylin and eosin (HE) staining was used for morphometric analysis, while Masson's staining distinguished the extracellular matrix (ECM) and smooth muscle fibres. Resorcin–Fuchsin (RF) staining was applied to highlight elastic fibres and the internal elastic lamina (IEL). External jugular veins from rats without graft implantation served as controls.

The luminal area was calculated by determining the space within the IEL perimeter, and the residual lumen was measured. The difference between these two values indicated the neointimal area. Regions of interest were quantified using computer‐assisted planimetry, analysing images captured by a colour digital camera (Nikon, DS‐L1) attached to a light microscope. NIH cell nuclei were manually traced at the highest magnification.

### 2.4 Immunohistochemical analysis

Paraffin‐embedded sections were deparaffinised and rehydrated. Antigen retrieval was conducted in a microwave using citrate buffer (pH 6). Endogenous horseradish peroxidase (HRP) activity was blocked with 3% H_2_O_2_. Following a 1‐h incubation in 10% horse serum at 37°C, sections were treated overnight at 4°C with primary antibodies (Table S1). After washing, secondary antibodies were applied for 60 min. Positive signals were visualised with a DAB Horseradish Peroxidase Color Development Kit, resulting in a reddish‐brown colour, and sections were counterstained with haematoxylin. ImageJ was utilised for quantitative analysis.

### 2.5 Immunofluorescence

As previously detailed,^30^ vascular sections were deparaffinised and rehydrated, and antigen retrieval was performed using citrate buffer (pH 6) in a microwave. After blocking with 5% donkey serum for 1 h at 37°C, primary antibodies (Table S2) were applied overnight at 4°C. Following multiple PBS washes, fluorescent secondary antibodies and DAPI were added for 1 h with rocking at room temperature. Samples were mounted in Fluoromount‐G (Southern Biotech). The immunofluorescence slides were placed in a digital tissue scanner (Pannoramic MIDI, 3DHISTECH). The scanning area was manually selected, and the appropriate fluorescence scanning channels were chosen (Dapi: 365 nm excitation, 420–470 nm emission; SpGreen: 484–504 nm, 517–537 nm; SpRed: 576–596 nm, 618–638 nm). Automatic focusing was performed, and each channel's exposure time was sequentially adjusted to maintain a black background and optimal fluorescence intensity. ImageJ was used for quantifying the relative levels.

### 2.6 Terminal deoxynucleotidyltransferase‐mediated dUTP nick‐end labelling (TUNEL) assay

A fluorescein (FITC) TUNEL Cell Apoptosis Detection Kit (G1501, Servicebio, Wuhan, China) was utilised to assess apoptotic cells. Briefly, deparaffinised and rehydrated sections were treated with Proteinase K (200 µg/mL) at 37°C for 20 min. Following several PBS washes, a permeabilisation solution (.1% Triton X‐100 in .1% sodium citrate) was applied at room temperature for 20 min. After incubation with equilibration buffer for 10 min, sections were treated with TdT incubation buffer containing recombinant TdT enzyme and FITC‐12‐dUTP Labeling Mix at 37°C for 1 h. After further washing, the sections were blocked with 5% donkey serum at 37°C for 1 h and then incubated overnight at 4°C with α‐SMA. Fluorescent secondary antibodies and DAPI were applied for 1 h with rocking at room temperature, and the samples were mounted in Fluoromount‐G (Southern Biotech).

### 2.7 ELISA assay

Serum levels of rat tumour necrosis factor (TNF)‐*α* were measured using a rat TNF‐*α* ELISA kit (E‐EL‐R2856, Elabscience, Wuhan, China), following the manufacturer's instructions. Three serum replicates were tested for each rat.

### 2.8 Slide preparation for Visium

Control veins and Day 19 graft veins were collected and embedded in OCT. Ten control veins were cryosectioned and assembled into a barcoded capture area labelled RatV0. Similarly, ten Day 19 graft veins were cryosectioned and assembled into three barcoded capture areas, labelled RatV1, RatV2, and RatV3 (Figure S2). These samples were biological replicates, as they were derived from 10 different rats. Each barcoded capture area was cryosectioned at a 10 µm thickness for Visium Spatial slides using the 10× Genomics Visium platform. Tissue sections from each area were collected, RNA was extracted according to the manufacturer's protocols, and RNA quality was assessed, yielding RIN values of 7.5‐8.8. Sections were placed on the Visium tissue optimisation slide and spatial gene expression slide. HE staining and bright‐field imaging were performed on the sections. Optimisation protocols were conducted as outlined in the guide. In brief, tissues underwent a time‐course permeabilisation, followed by reverse transcription, generating fluorescent cDNA. Fluorescence imaging was conducted using a Texas Red filter set, and a 30‐min permeabilisation time was determined to yield the highest fluorescence signal with minimal diffusion.

### 2.9 Visium workflow library preparation and sequencing

The sections were permeabilised for 30 min to release mRNA from the overlying cells onto the capture areas. First‐strand cDNA synthesis was carried out via reverse transcription, followed by second‐strand synthesis. The cDNA was then transferred from the slide to a PCR tube, where spatially barcoded, full‐length cDNA was amplified through PCR to generate sufficient material for library construction. During this process, P5, P7, i7, and i5 sample indexes, along with TruSeq Read 2, were added through end repair, A‐tailing, adaptor ligation, and additional PCR. The final libraries containing P5 and P7 primers were used for amplification on an Illumina platform. Libraries were sequenced on a NovaSeq 6000 using a custom paired‐end sequencing protocol. The Visium Spatial library comprised standard Illumina paired‐end constructs, with the 16 bp spatial barcode and 10 bp unique molecular identifier (UMI) encoded in Read 1, and sample index sequences incorporated as the i7 index read.

### 2.10 Alignment and Unique Molecular Identifier (UMI) counting of Visium data

Space Ranger employed image processing algorithms to determine tissue placement and align the printed fiducial spot pattern. Tissue detection identified capture spots and their associated barcodes, while fiducial alignment enabled Space Ranger to locate individual barcode points within the image. The STAR (Spliced Transcripts Alignment to a Reference) aligner was then used to map reads to the genome, utilising transcript annotation (GTF) for accurate read alignment. Only mapped reads were considered for UMI counting. Reads mapped to the transcriptome were grouped by shared barcode, UMI, and gene annotation, with the number of unique UMIs representing the expression level of the gene in each spot.

### 2.11 Quality analysis

Spots overlying no tissue, distorted tissue, or folded sections were excluded from the final analysis. A total of 440, 573, 380, and 744 spots from RatV0, RatV1, RatV2, and RatV3, respectively, were included in the analysis (Tables S3 and S4).

### 2.12 Clustering spots

Supervised clustering was conducted for the spot clusters. Due to the thin endothelial cell layer in the control group veins, distinguishing features were difficult to identify. Two layers, control‐med (media) and control‐adv (adventitia), were defined based on the boundary between the media and adventitia in the control veins. One control external jugular vein sample was excluded because the boundary of the middle layer could not be determined. The D19 graft was divided into D19‐int (intima), D19‐med (media), and D19‐adv (adventitia) based on histological analysis. One D19 sample was also removed due to sectioning issues. Spots corresponding to control‐med, control‐adv, D19‐int, D19‐med, and D19‐adv were merged into their respective subsets for comparative analysis.

### 2.13 Principal component analysis (PCA) and transcriptome proximity analysis

PCA was performed to evaluate the differences between D0 and D19 spots, using the RunPCA function from the Seurat package (Version 3.1.5). As a multivariate statistical method, PCA examines correlations among multiple variables by transforming the original indicators into a linear combination of new composite indicators. This technique condenses correlated data points into a few uncorrelated variables, known as principal components, optimising the dispersion of individual data points in an unbiased manner. This process enables the identification of distinct clusters, which are graphically represented based on their positions on the PCA plot when two principal components are displayed. Variable genes served as input for PCA, with significant principal components identified through the jackstraw method. The top 2000 genes displaying the highest variation across cells were selected, and the ScaleData function in Seurat was applied to standardise the expression data. Following dimensionality reduction, the principal components were ranked according to the variance explained by each, and the first two components were extracted to generate a scatter plot using the DimPlot function. A modified Calinski–Harabasz descriptor was employed to quantify the degree of separation between the two groups. The calculation formula was shown in Supplement method. 1.

To further quantify the similarity between the different spots in Control‐med, D19‐int, D19‐med, D19‐adv, and Control‐adv, the Pearson's correlation coefficient was computed for all expressed genes using the corr.test function from the R base package. Visualisation was performed with the corrplot package (Version 0.85). The correlation coefficient between replicates was also calculated to assess sample repeatability, with values closer to 1 indicating higher reproducibility between parallel experiments.

### 2.14 Differentially expressed gene (DEG) analysis

Model‐based Analysis of Single‐cell Transcriptomics (MAST) was applied to identify DEGs across predefined anatomical regions within the tissue. MAST excels in both supervised analyses of gene and gene module differential expression and unsupervised analyses of model residuals to propose hypotheses regarding gene co‐expression. It effectively models the bimodal distribution typical of single‐cell data by jointly estimating the expression rate and positive mean values. By integrating data from both discrete and continuous components, MAST facilitates the inference of changes in gene expression using statistics at the gene or gene set level.

DEGs were considered significant based on the following criteria: adjusted *p*‐value ≤ .05, log2FC ≥ 1, and a detection rate of > 0% within specific clusters. To highlight gene expression specificity across different tissue regions, certain genes were selected as markers based on differential expression results for each region. The expression profiles of these marker genes were visualised through heatmaps (ComplexHeatmap v2.4.3) and volcano plots (Enhanced Volcano v1.6.0). Upregulated DEGs were listed in ascending order of adjusted *p* values (Table S5).

### 2.15 GO and KEGG enrichment analyses of DEGs

GO and KEGG pathway analyses were conducted on all DEGs, which were filtered based on their biological functions. Functional enrichment analysis was performed using GO (http://www.geneontology.org/) and KEGG (http://www.genome.jp/kegg/) with the ‘clusterProfiler’ package (v3.16.1) based on a hypergeometric distribution.

The GO gene sets, covering molecular function, biological process, and cellular component categories, served as the reference for analysis. GO enrichment analysis identified all significantly enriched GO terms in peak‐related genes compared to the genome background, filtering genes based on their corresponding biological functions. All peak‐related genes were mapped to GO terms in the Gene Ontology database, and gene counts were calculated for each term. Significantly enriched GO terms were determined using a hypergeometric test. Similarly, KEGG, a major public database for pathway‐related information, was used for pathway enrichment analysis, identifying significantly enriched metabolic or signal transduction pathways in peak‐related genes compared to the whole genome background, following the same calculation method. Significantly enriched GO terms and KEGG pathways were identified among DEGs using a multiple test correction threshold of *p* < .05 (Holm–Bonferroni method). This approach effectively pinpointed the key biological functions associated with DEGs, allowing for the filtering of DEGs based on their specific biological roles.

### 2.16 Ex vivo static HSV culture and adenoviruses‐mediated FHL1 gene overexpression in HSV assay

HSV samples were collected in accordance with the guidelines of the Medical Ethics Committee of The First Affiliated Hospital of Sun Yat‐Sen University, with approval from the IEC for Clinical Research and Animal Trials (Approval number: [2023]826). Discarded HSV segments were obtained from the lower limbs of patients undergoing coronary artery bypass graft surgeries at the same hospital. This study involved samples from three different patients. Using sterile techniques, the discarded HSV segments were washed with cold PBS, and each segment was subsequently cut into three rings, each 2.0 mm in width. These rings were then randomly assigned to different experimental conditions (D0; D14(Ad‐NC); D14(Ad‐FHL1)). The D0 group served as the baseline. Adenoviruses for the empty virus (Ad‐NC) and FHL1 overexpression (Ad‐FHL1) were synthesised by HanBio (Shanghai, China). To determine the optimal adenovirus infection efficiency, HSV rings were infected with four concentrations: 1$\times 10^{10}$ PFU/mL, 4$\times 10^{10}$ PFU/mL, 7$\times 10^{10}$ PFU/mL, and 1$\times 10^{11}$ PFU/mL. Two experimental groups were pretreated with either Ad‐FHL1 (HBAD‐Adeasy‐h‐FHL1‐3xflag‐EGFP) or Ad‐NC (HBAD‐EGFP) at 7$\times 10^{10}$ PFU/mL for 12 h, followed by 14 days of culture in RPMI 1640 medium supplemented with 30% fetal bovine serum and 1% antibiotic solution at 37°C under 5% CO_2_. The culture medium was refreshed every 72 h. After the culture period, pathological analysis of the HSV rings was performed as previously described.

The sequence information of FHL1 inserted into the adenovirus is provided in Supplementary Method 1.

### 2.17 Statistical analyses

Statistical analysis was conducted using Prism version 8.0. Analysts were blinded to group allocation during the analysis process. A pathologist, also blinded to sample identity, performed the quantification of histological, IHC, and IF measurements. Data normality was evaluated using the Pearson's normality test (α = .05). For comparisons between two groups, a two‐tailed Student's *t*‐test was used for normally distributed data, while the Mann‐Whitney test was employed for nonnormally distributed data. For comparisons involving multiple groups, one‐way ANOVA was applied to normally distributed data, and the Kruskal–Wallis test was used for nonnormally distributed data. Results are presented as mean ± standard deviation (SD), with each point representing an individual sample. Statistical significance was defined as *p* < .05. No formal power calculation was conducted for sample size determination; however, based on prior experience, a sample size of at least five per group was deemed sufficient to achieve significance in the rat model and ST analysis. Statistical significance levels are denoted as *p < .05, **p < .01, ***p < .001, and ****p < .0001.

## 3 RESULTS

### 3.1 In histological analysis, newly generated *α*‐SMA(+) cells outside the grafted vein repaired the vein graft media and contributed to NIH

**FIGURE 1:**
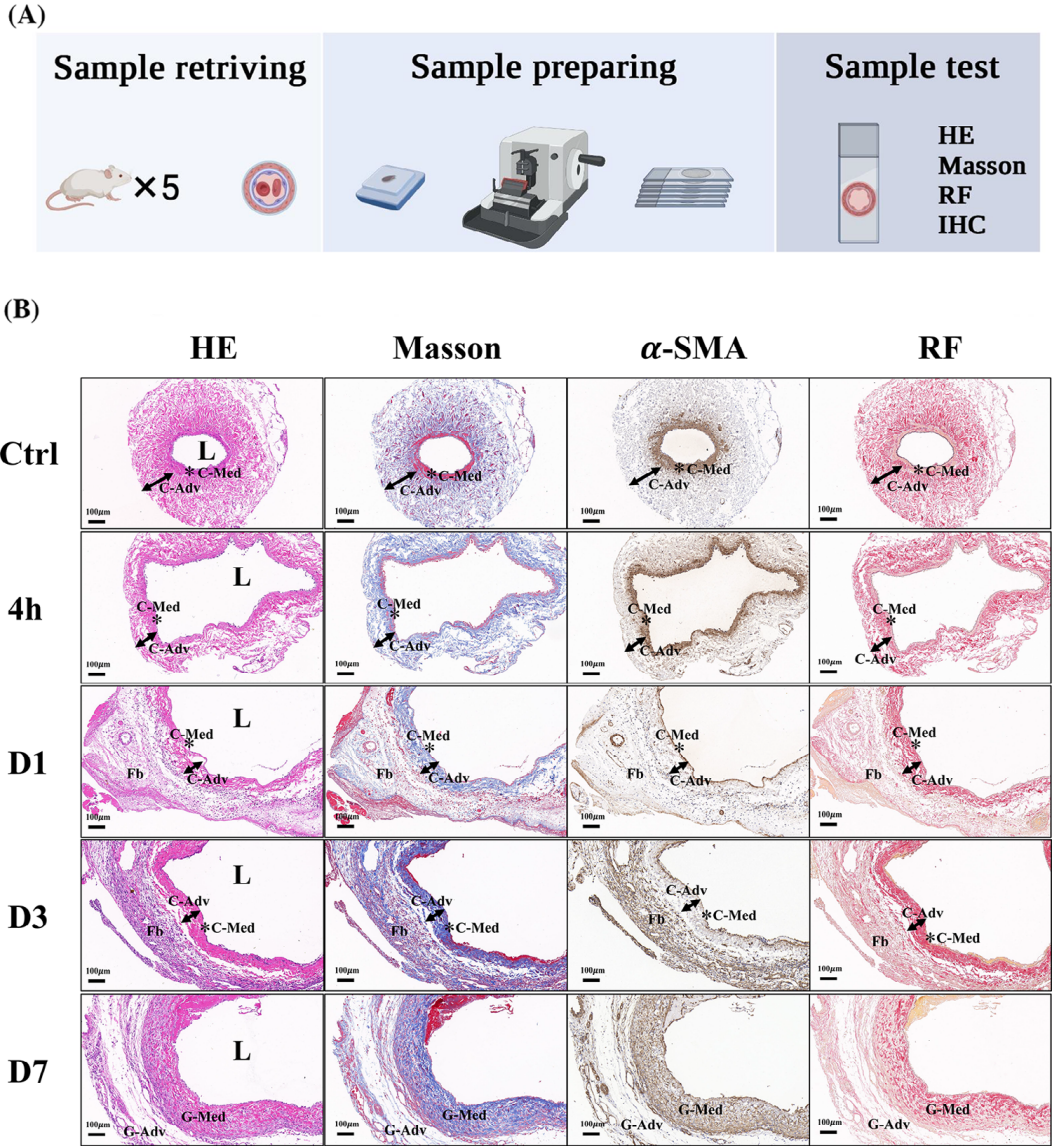
Time‐course analysis of NIH during the nascent‐NIH phase in vein grafts. (A) Schematic representation of the experimental procedure (*n* = 5 rats per time point). (B) HE, Masson, Resorcin–Fuchsin (RF), and *α*‐SMA immunohistochemistry (IHC) staining of vein grafts at sequential time points (control, hour 4, Day 1, Day 3, and Day 7) during the nascent‐NIH phase (*n* = 5 rats per time point). C‐Med: media tunica of the control external jugular vein; C‐Adv: adventitia of the control external jugular vein; Fb: fibrin; G‐Med: media tunica of the vein graft; G‐Adv: adventitia of the vein graft; L: lumen. Scale bar = 100 µm.

**FIGURE 2:**
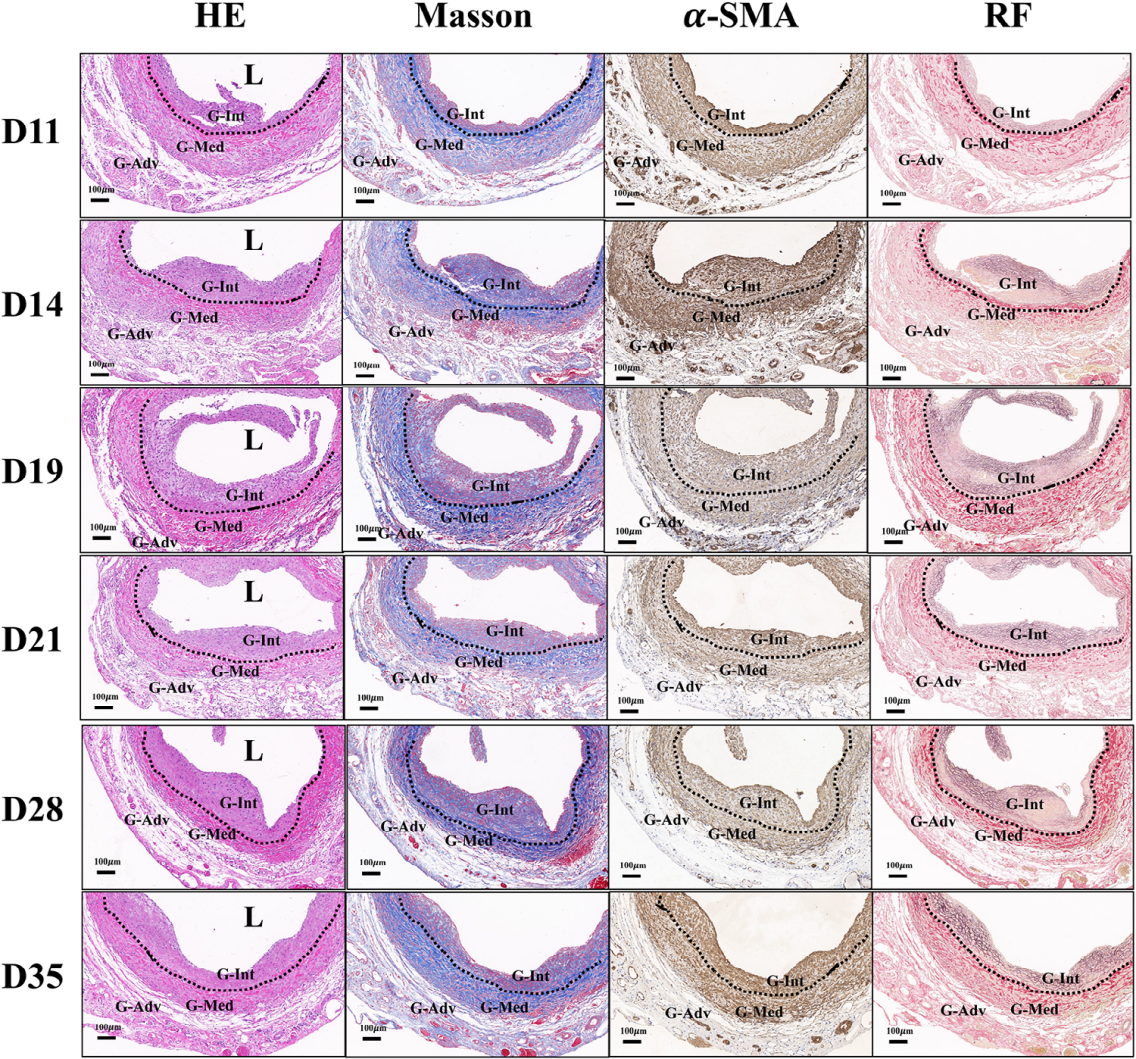
Time‐course analysis of NIH during the NIH phase in vein grafts. HE, Masson, RF, and *α*‐SMA IHC staining of vein grafts at consecutive time points (Day 11, Day 14, Day 19, Day 21, Day 28, and Day 35) during the NIH phase (*n* = 5 rats per time point). G‐Int: neointima of the vein graft; G‐Med: media tunica of the vein graft; G‐Adv: adventitia of the vein graft; L: lumen. Dotted line represents the internal elastic lamina. Scale bar = 100 µm.

To morphologically assess NIH processes, HE, Masson, Resorcin–Fuchsin (RF), and immunohistochemical staining were performed on vein grafts at 11 different time points (Figure 1A). The remodelling process was divided into two distinct phases: the nascent‐NIH phase (0‐10 days) (Figure 1B) and the NIH phase (11‐35 days) (Figure 2), based on the formation of typical neointima.

**FIGURE 3:**
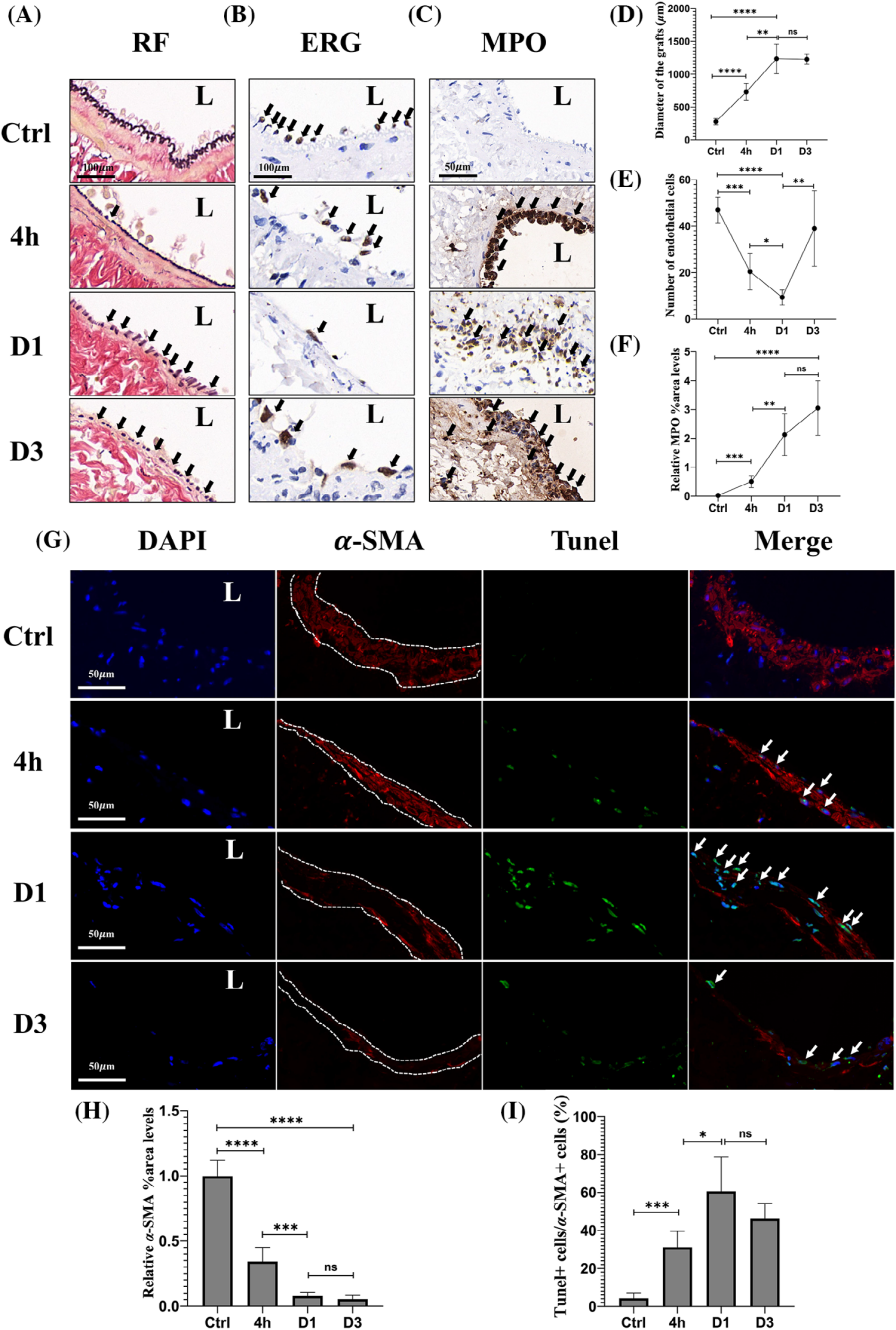
Cellular injury in vein grafts following grafting. (A) Resorcin–Fuchsin staining shows disruption of the internal elastic lamina (IEL) due to vessel distension, indicated by arrows. Scale bar = 100 µm. (B) IHC analysis demonstrates endothelial cell (ERG+) damage and subsequent restoration after grafting, with arrows marking ERG‐stained endothelial cells. Scale bar = 100 µm. (C) IHC analysis reveals inflammation (MPO+), indicated by arrows highlighting neutrophils stained by MPO. Scale bar = 50 µm. Panels A–C, *n* = 5 rats per time point; L: lumen. (D) Line graph depicting changes in vein graft diameter. (E) Line graph showing the number of endothelial cells over time. (F) Line graph of MPO staining intensity in vein grafts. (G) Representative immunofluorescence (IF) images showing DAPI (blue), *α*‐SMA (red), and TUNEL (green) staining in vein grafts at control, hour 4, Day 1, and Day 3 (*n* = 5 rats per time point). The merged image highlights apoptotic smooth muscle cells (arrow). L: lumen. Dotted line indicates the medial layer structure. Scale bar = 50 µm. (H) Quantification of IF staining for medial α‐SMA‐expressing areas in vein grafts. (I) Proportion of TUNEL(+) cells relative to α‐SMA‐expressing cells in vein grafts.

**FIGURE 4:**
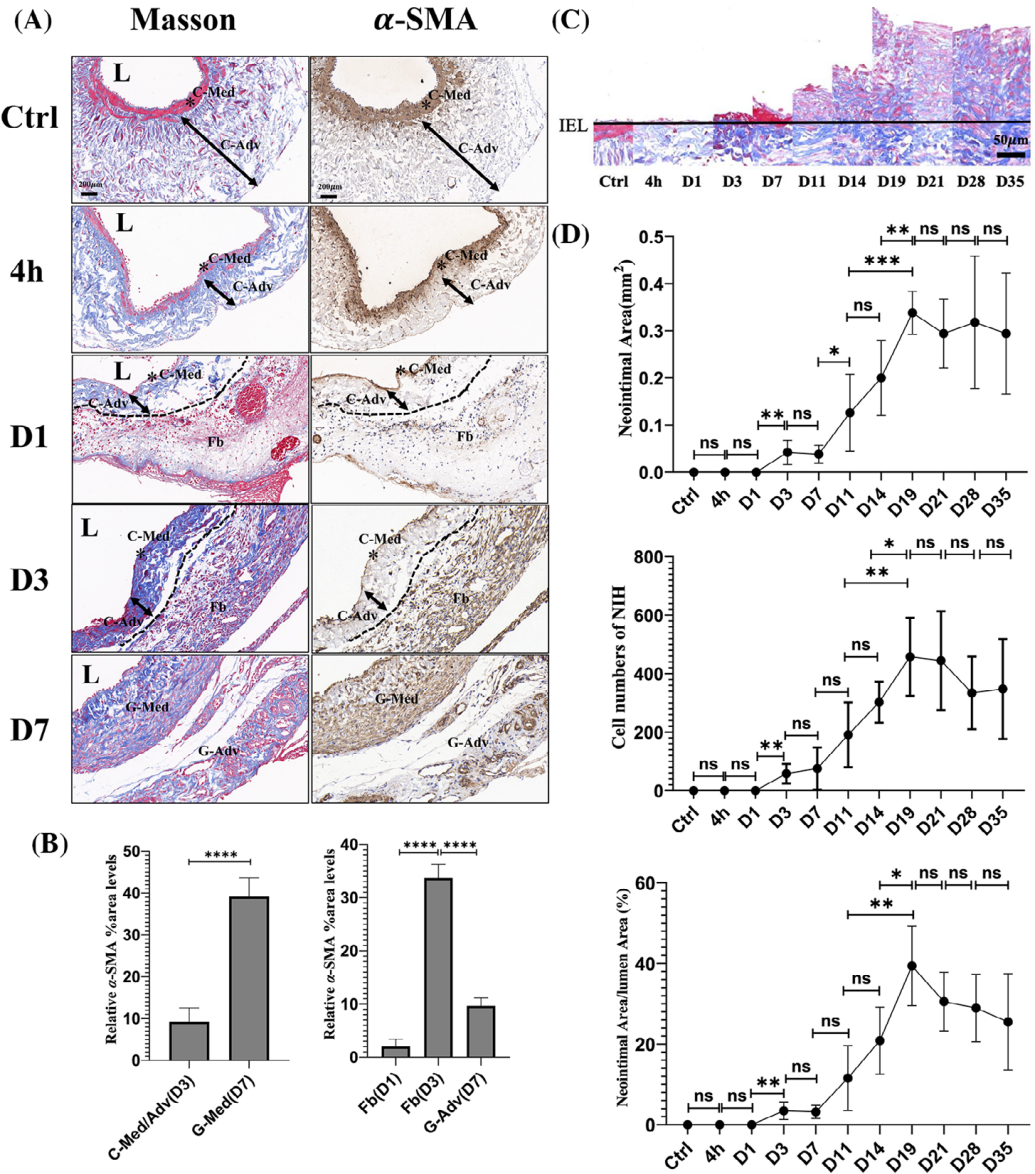
Cellular repair in vein grafts postgrafting. (A) Masson and *α*‐SMA IHC staining illustrating boundary changes and the presence of repair cells in vein grafts at different time points (control, hour 4, Day 1, Day 3, and Day 7) (*n* = 5 rats per time point). The dotted line marks the boundary of the control external jugular vein after arterial grafting. C‐Med: media tunica of the control external jugular vein; C‐Adv: adventitia of the control external jugular vein; Fb: fibrin; G‐Med: media tunica of the vein graft; G‐Adv: adventitia of the vein graft; L: lumen. Scale bar = 200 µm. (B) Quantification of α‐SMA IHC staining across different spatial regions at consecutive time points. (C) Schematic representation of neointimal thickness based on Masson staining across time points. Scale bar = 50 µm. Line represents the internal elastic lamina (IEL). (D) Changes in the neointimal area at different time points (top panel). Neointimal cell count at consecutive time points (middle panel). Neointimal area‐to‐lumen area ratio at consecutive time points (bottom panel). *n* = 5 rats per time point. Statistical analysis indicated a significant difference between Day 19 and Day 14, showing that neointimal formation peaked on Day 19. The luminal area within the neointima was measured by calculating the area enclosed by the IEL. *p* Values correspond to one‐way ANOVA with Tukey's multiple comparisons test for D, E, F, H, I, K (right panel), M; and two‐tailed unpaired *t‐*test for K (left panel). ns: not significant; *p < .05, **p < .01, ***p < .001, ****p < .0001.

During the nascent‐NIH phase, the normal external jugular vein, upon immediate exposure to high arterial pressure, expanded to five times its normal diameter, and the internal elastic lamina (IEL) became discontinuous and ruptured (Figure 3A and D). Endothelial cells in the grafted veins were extensively denuded, with their numbers reduced to only 20% of those in normal veins (Figure 3B and E). Neutrophils transitioned from adhering to the endothelial layer to infiltrating the full thickness of the graft (Figure 3C and F). The medial SMCs in the normal jugular vein were continuous and intact; however, following grafting into the artery, apoptosis occurred (TUNEL+ SMCs), and the majority of medial SMCs had disappeared by day 3. In grafted veins, *α*‐SMA expression was reduced to only 5% of normal levels, leaving an acellular medial layer with an indistinct boundary from the adventitia (Figure 3G, H, and I). These results indicate that, under the influence of various injury factors, the structural integrity of all vessel layers was severely compromised, with medial SMCs undergoing apoptosis and largely disappearing by the third day. Subsequently, during the repair phase, the adventitia of the normal jugular vein, initially composed of clearly defined collagen fibres (blue in Masson staining), exhibited no significant changes at 4 h postgrafting. However, by day 1, Masson staining revealed the formation of a bright red cellulose layer outside the adventitia, constituting a new boundary for the graft. Few *α*‐SMA(+) cells were observed in this layer on the first day, but by day 3, a large number of *α*‐SMA(+) cells had populated the region. By day 7, the *α*‐SMA(+) cells in the cellulose layer had disappeared, and a newly formed adventitial layer was observed. Additionally, numerous *α*‐SMA(+) cells emerged at the location where the acellular medial layer fused with the adventitia of the original vein, contributing to the formation of a new medial layer within the grafts (Figure 4A and B). Histological analysis of these dynamic morphological changes suggests that newly generated *α*‐SMA(+) cells external to the jugular vein played a key role in reconstructing the medial layer in the grafts.

During the NIH phase, a typical neointima, characterised by protrusion from IEL and extracellular matrix deposition, began to form on day 11. A moderate amount of cellulose accumulated within the lumen (Figure S3). At this stage, nascent *α*‐SMA(+) cells exhibited amoeboid movements and nuclear deformation as they traversed the ruptured IEL (Figure S4). Histological evidence suggests that these cells migrated from the media to the neointima. Neointimal growth accelerated from day 11, peaking on day 19, after which NIH development plateaued and remained stable (Figure 4C and D).

### 3.2 The phenotype of neointimal cells was between that of SMCs and fibroblasts

**FIGURE 5:**
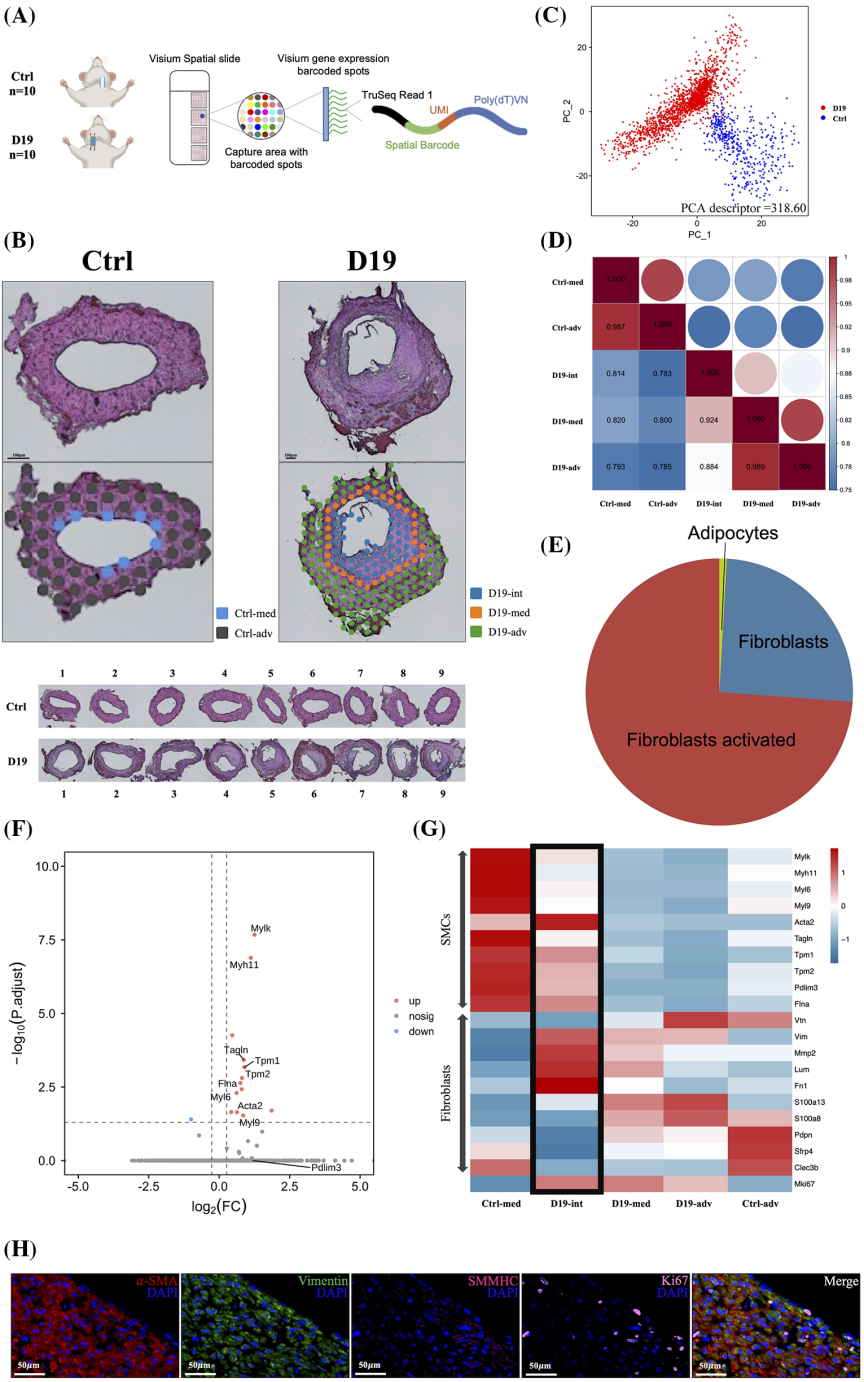
Spatial transcriptomics atlas and gene expression profiling in control and Day 19 graft veins. (A) Workflow schematic for spatial transcriptomics (ST). (B) The final analysis included nine control veins and nine Day 19 vein grafts. ST spots from unbiased clustering were overlaid on each vessel (biologically independent). Ctrl‐med: media tunica of the control external jugular vein; Ctrl‐adv: adventitia of the control external jugular vein; D19‐int: neointima of the Day 19 vein graft; D19‐med: media tunica of the Day 19 vein graft; D19‐adv: adventitia of the Day 19 vein graft. Scale bar = 100 µm. (C) Principal component analysis revealed significant deviations in gene expression profiles between control veins and Day 19 vein grafts, with PCA descriptors used for statistical measurement. (D) Transcriptome proximity analysis comparing spatial regions between control veins and Day 19 grafts, with the Pearson's correlation coefficient quantifying statistical correlations. (E) Representative pie chart showing cell‐type proximity maps and associated cell populations in spatial RNA‐seq data. (F) Volcano plot highlighting key DEGs (*Mylk*, *Myh11*, *Tagln*, *Tpm1*, *Tpm2*, *Flna*, *Myl6*, *Myl9*, *Pdlim3*, and *Acta2*) associated with SMC contractile markers identified in the tunica media versus the adventitia of control veins. (G) Heatmap depicting gene expression profiles for SMCs, fibroblasts, and proliferation markers in annotated regions across different layers of control veins and Day 19 grafts. The black frame highlights the neointima expression profile in the Day 19 graft. (H) Representative immunofluorescence images showing DAPI (blue), *α*‐SMA (red), Vimentin (green), SMMHC (rose red), and Ki67 (pink) staining in the neointima of Day 19 vein grafts. Scale bar = 50 µm.

To enhance the understanding of NIH, ST was applied to profile gene expression patterns in control veins and day 19 grafts. Normal external jugular veins from 10 rats served as the control group, while grafts from 10 rats, 19 days postoperation, constituted the D19 group (Figure 5A). Following imaging quality control, spots not meeting the predefined criteria were excluded, and nine vessels from each group were included in the final analysis (Figure S2). Each sample exhibited valid spatial barcode detection rates exceeding 97%, with sequencing saturation levels surpassing 93% (Table S3), confirming high data quality and reliability. Analysis of 2137 spots within the capture region yielded an average detection of 1698 genes per spot, with a cumulative total of 15 268 genes (Table S4). A supervised clustering method was employed to classify vessels into distinct clusters based on histological spatial regions. Due to the thin endothelial cell layer, veins in the control group were divided into two clusters: Ctrl‐med and Ctrl‐adv. In contrast, the D19 grafts were segmented into three clusters: D19‐int, D19‐med, and D19‐adv (Figure 5B).

PCA demonstrated a clear separation between the control group (normal veins) and the D19 group (19‐day grafts). The PCA descriptor, reflecting the degree of differentiation between the two groups, was 318.60 (identical images = 0, poor separation < .3, and good separation ≥ 1) (Figure 5C). Transcriptomic proximity between different spatial regions of the two groups was subsequently assessed using Pearson's correlation coefficients. The D19 grafts exhibited weak similarity to the control group in each spatial region (.783 < *r* < .820). In contrast, within the D19 group, the spatial proximity showed strong similarity among the three layers, while spatial distance displayed weak similarity (*r* = .989 for adventitia and media, *r* = .924 for intima and media, and *r* = .884 for adventitia and intima) (Figure 5D). These findings from PCA and transcriptome proximity analysis revealed substantial transcriptomic differences between the control and D19 groups. Despite strong spatial similarities within the D19 group, some transcriptomic distinctions persisted among spatial regions.

Gene markers were used to annotate and classify the sequencing data via the SingleR package, revealing a cell population dominated by stromal cells, primarily activated fibroblasts, fibroblasts, and adipocytes (Figure 5E). In the control group, the media consisted of contractile SMCs, while the adventitia was composed of noncontractile fibroblasts. Differential gene expression analysis between the Ctrl‐med and Ctrl‐adv clusters identified key DEGs related to the contractile phenotype, including Mylk, Myh11, Tagln, Tpm1, Tpm2, Flna, Myl6, Myl9, Pdlim3, and Acta2 (Figure 5F). Based on these DEGs, the phenotype of neointimal cell clusters in the D19 group was characterised. Neointimal cells demonstrated a hybrid expression profile, predominantly expressing fibroblast‐associated genes (Vtn, Vim, Mmp2, Lum, Fn1, S100a13, S100a8, Pdpn, Sfrp4, Clec3b) and proliferation markers (Mki67), while also exhibiting contractility‐related genes (Mylk, Myh11, Tagln, Tpm1, Tpm2, Flna, Myl6, Myl9, Pdlim3, Acta2) (Figure 5G). Immunofluorescence further confirmed the presence of nonmature muscle marker α‐SMA, fibrosis marker Vimentin, and proliferative marker Ki67, alongside weak expression of the mature muscle marker SMMHC (Figure 5H). However, in later stages, neointimal cells re‐expressed mature contractile markers such as SMMHC and Cnn1 (Figure S5). These results indicate that cells in the neointimal layer exhibit a hybrid phenotype between fibroblasts and SMCs, with proliferative characteristics suggestive of an activated fibroblast state.

### 3.3 Functional enrichment analysis of DEGs in the neointima

**FIGURE 6:**
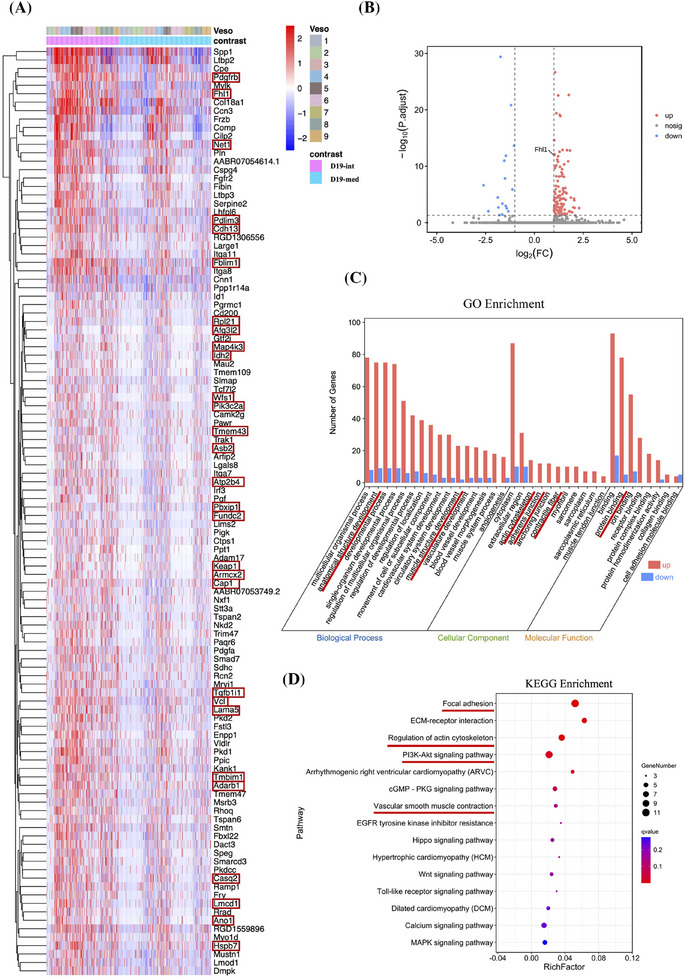
Differentially expressed genes (DEGs) in the neointima and GO/KEGG analyses. (A) Heatmap showing upregulated DEGs in the neointima (adjusted *p* ≤ .05; fold change ≥ 2). The red frame marks the 29 upregulated DEGs selected for IHC validation. (B) Volcano plot of DEGs in the neointima. (C) GO analysis of DEGs clustered into biological process (blue), cellular component (green), and molecular function (orange), based on the Cluster Profiler package in R software. (D) KEGG pathway enrichment analysis illustrating the primary biological functions of the DEGs.

Although the three spatial regions of the grafts in the D19 group exhibited strong similarities, differential gene expression analysis between the neointima and media was performed to identify variations within the neointima across the entire graft. A total of 130 DEGs were identified, with 110 genes significantly upregulated and 20 downregulated in the neointima compared to the media (Figure 6A and B). The upregulated genes are displayed in a heatmap for visual comparison.

To elucidate the functions of these DEGs, GO biological function enrichment analysis was conducted, mapping the DEGs to biological processes, cellular components, and molecular functions. These genes were significantly enriched in three primary functional clusters: (1) biological processes related to cardiovascular and muscle development; (2) cellular components such as the actin cytoskeleton, contractile fibres, myofibrils, sarcomeres, sarcoplasmic reticulum, and muscle‐tendon junctions; and (3) molecular functions involved in binding (Figure 6C). Additionally, KEGG pathway enrichment analysis highlighted significant enrichment in pathways including focal adhesion, actin cytoskeleton regulation, the PI3K‐Akt signalling pathway, and vascular smooth muscle contraction (Figure 6D). Network analysis of the pathways and associated genes was also performed (Figure S6).

### 3.4 Fhl1 was stably and specifically expressed in the neointima of vein grafts

**FIGURE 7:**
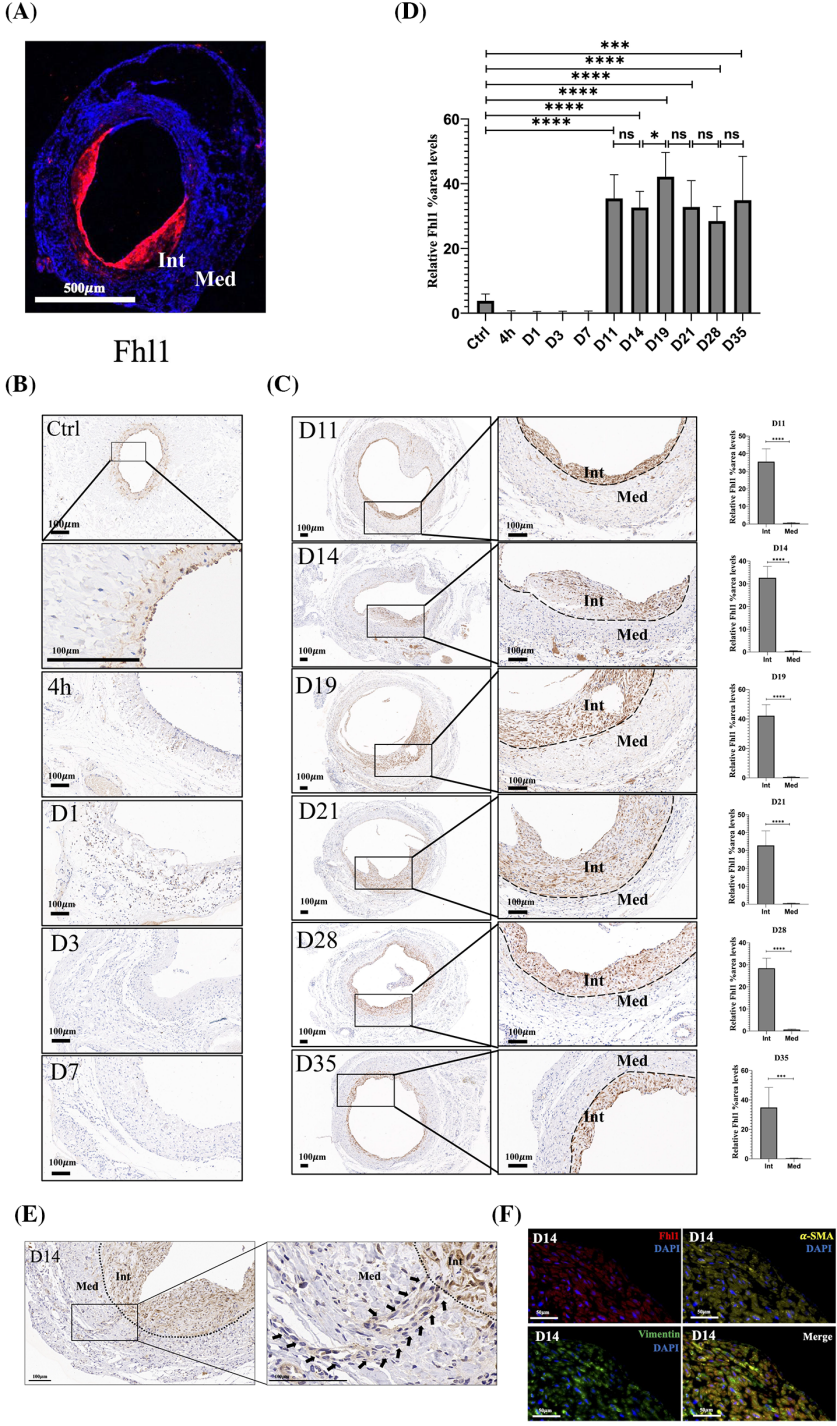
Time course of Fhl1 expression in the neointima of vein grafts. (A) IF staining of Fhl1 protein expression in Day 19 vein grafts. Scale bar = 500 µm. (B) IHC staining of Fhl1 protein expression during the nascent‐NIH phase. Scale bar = 100 µm. (C) IHC staining of Fhl1 protein expression during the NIH phase (left panel). Scale bar = 100 µm. Quantification of Fhl1 expression in the neointima versus the media (right panel). Int: neointima of vein graft; Med: media tunica of vein graft; dotted line indicates the IEL. (D) Quantification of Fhl1 expression in the neointima over consecutive time points. Panels B–D, *n* = 5 rats per time point. (E) IHC staining of Day 14 vein grafts showing cells migrating from the tunica media to the neointima and beginning to express Fhl1 protein (arrow). (F) Representative IF images of DAPI (blue), Fhl1 (red), *α*‐SMA (yellow), and Vimentin (green) staining in the neointima of grafts. Scale bar = 50 µm. *p*‐values correspond to two‐tailed unpaired *t*‐test for panel C; one‐way ANOVA with Tukey's multiple comparisons test for panel D. ns: not significant; *p < .05, ***p < .001, ****p < .0001.

Based on morphological observations and GO/KEGG analyses, the upregulated genes are likely involved in regulating the cytoskeleton, muscle development, and adhesion processes. From previous studies, 29 upregulated DEGs were selected for further examination (Figure 6A), and their spatial expression patterns were analysed within the D19 group (Figures S7‐S9). The spatial distribution of these genes in the media and neointima was validated using immunohistochemical staining of graft sections from day 19 (Figure S10). Notably, Fhl1 was almost exclusively enriched in neointimal cells, whereas the other genes lacked specific spatial enrichment (Figure 7A). To further assess the spatial specificity and stability of Fhl1 expression throughout the entire vein graft remodelling process, Fhl1 immunohistochemical staining was conducted at all time points. Fhl1 was weakly expressed in the media of control veins, with negligible expression during the nascent‐NIH phases (Figure 7B). However, during the NIH phases, Fhl1 expression was specific to the neointimal region, remaining strong and stable throughout (Figures 7C and D and S11). Histological analysis also identified a subset of cells that appeared to migrate from the media to the neointima; these cells exhibited weak Fhl1 expression in the media but began expressing Fhl1 robustly after crossing the IEL into the neointima (Figure 7E). Furthermore, Fhl1 co‐localised with both *α*‐SMA and Vimentin within the neointima (Figure 7F). These results indicate that Fhl1 expression, consistent with the hybrid phenotype of neointimal cells, aligns with the NIH phases and is stably and specifically localised within the neointima. Thus, Fhl1 may play a pivotal role as a regulator of NIH.

### 3.5 Genetic ablation of Fhl1 in vivo exacerbated NIH

**FIGURE 8:**
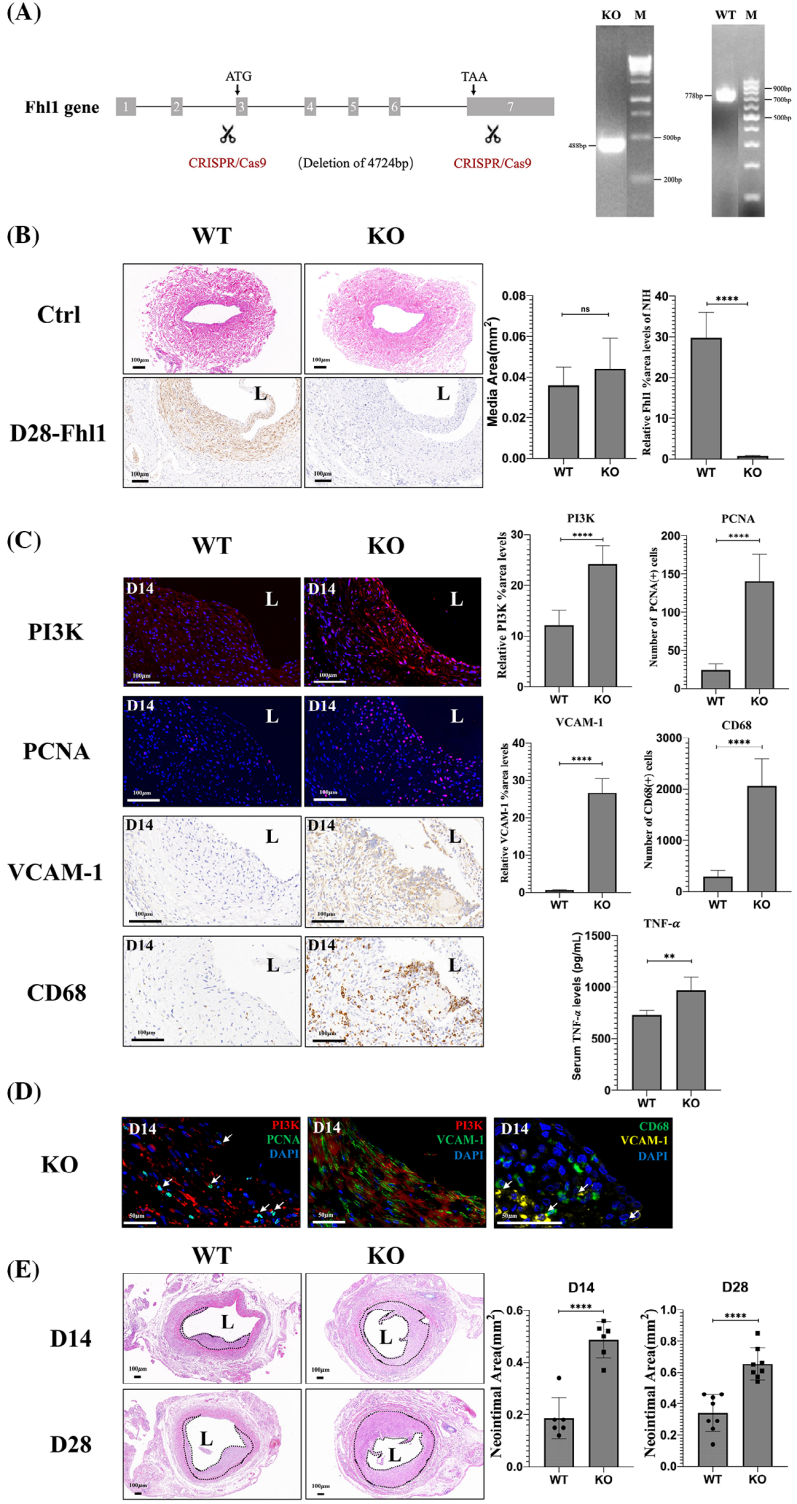
Fhl1 deficiency exacerbates neointima formation in vivo. (A) Strategy for CRISPR/Cas9‐mediated genome editing of the Fhl1 gene (left panel). Genotype detection by PCR using rat tail snip (right panel). (B) HE staining of the external jugular vein and Fhl1 expression detected by IHC in Day 28 vein grafts from WT and Fhl1‐KO rats (*n* = 5 rats per group) (left panel). Quantification of the media area of the external jugular vein and Fhl1 average staining intensity in the neointima of Day 28 grafts (right panel). (C) Representative images (left) and quantification (right) of IF or IHC for PI3K, PCNA, VCAM‐1, CD68 in the grafts, and serum TNF‐*α* levels in WT and Fhl1‐KO rats at Day 14 postgrafting (*n* = 6 rats per group). (D) Representative IF images of DAPI (blue), PI3K (red), PCNA (green), PI3K (red) with VCAM‐1 (green), and CD68 (green) with VCAM‐1 (yellow) staining in the neointima of Fhl1 KO grafts. Scale bar = 50 µm. (E) Cross‐sections of HE‐stained vein grafts (left) and quantification of neointima area (right) in WT and Fhl1‐KO rats at Day 14 (*n* = 6 rats per group) and Day 28 (*n* = 8 rats per group) postgrafting. Dotted area represents the neointima. L: lumen. Scale bar = 100 µm. *p*‐values correspond to two‐tailed unpaired *t*‐test for panels B–E. ns: not significant; **p < .01, ****p < .0001.

To investigate the in vivo function of Fhl1, CRISPR/Cas9 technology was employed to generate Fhl1 KO rats. Genotyping was confirmed through PCR analysis of rat tail snips, followed by vein graft surgery on Fhl1 homozygous KO rats (Figure 8A). Fhl1 expression was notably high in skeletal muscle. Histological examination revealed no structural differences in the external jugular vein or skeletal muscle between Fhl1 KO and WT rats under baseline conditions. However, in grafts and skeletal muscle of KO rats, Fhl1 protein expression was significantly reduced (Figures 8B and S12). Compared to WT grafts, Fhl1 KO grafts exhibited increased activation of the phosphoinositide 3‐kinase (PI3K) pathway, elevated cell proliferation, higher expression of the inflammatory adhesion molecule VCAM‐1, and an increased number of infiltrating CD68(+) macrophages (*p* < .0001). The absence of Fhl1 also led to a marked rise in the serum levels of TNF‐*α*, a key inflammatory cytokine (Figure 8C). In terms of spatial distribution, Fhl1 KO grafts showed co‐localisation of PI3K with PCNA, and PI3K with VCAM‐1. Additionally, VCAM‐1 and CD68 exhibited spatial proximity (Figure 8D). As a result, neointimal area in Fhl1 KO rats was significantly larger than in WT rats at both 14 and 28 days postsurgery (D14: WT vs. KO: .19 ± .08 mm^2^ vs. .49 ± .07 mm^2^, *n* = 6 per group, *p* < .0001; D28: WT vs. KO: .34 ± .12 mm^2^ vs. .65 ± .10 mm^2^, *n* = 8 per group, *p* < .0001) (Figure 8E). Moreover, 28 days postsurgery, the medial structure of the grafts in Fhl1 KO rats was significantly reduced (Figure S13).

Late‐stage neointimal cells from Fhl1 KO rats at day 28 also expressed mature contractile markers such as SMMHC (Figure S14). These results suggest that Fhl1 genetic ablation leads to heightened proliferation and inflammatory responses in grafts, resulting in increased neointima formation. Additionally, the spatial distinction between neointimal and medial cells could not be maintained.

### 3.6 Therapeutic FHL1 overexpression prevented NIH in HSV segments

**FIGURE 9:**
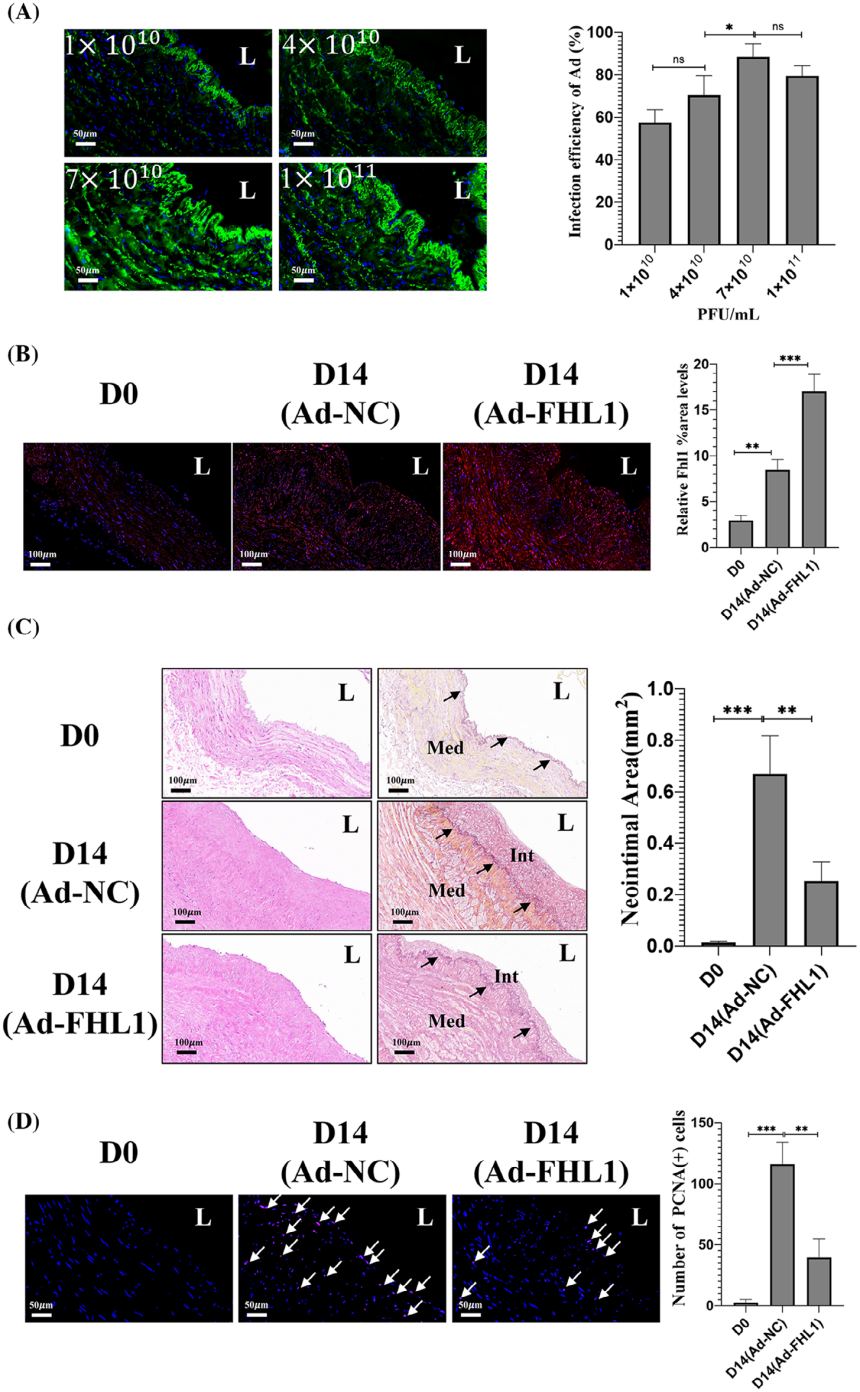
FHL1 overexpression inhibits NIH in cultured human saphenous veins (HSV). (A) Representative images (left) and quantification (right) of EGFP fluorescence in HSV rings infected with varying concentrations of virus: 1$\times 10^{10}$ PFU/mL, 4$\times 10^{10}$ PFU/mL, 7$\times 10^{10}$ PFU/mL, and 1$\times 10^{11}$ PFU/mL (*n* = 3 HSV rings per group). (B) IF detection of FHL1 expression in Day 14 HSV rings from the Ad‐NC and Ad‐FHL1 groups (left panel). Quantification of FHL1 regional staining intensity in Day 14 HSV rings (*n* = 3 HSV rings per group) (right panel). (C) Cross‐sections of HE and RF‐stained HSV rings (left) and quantification of neointima area (right) in Ad‐NC and Ad‐FHL1 groups on Day 14 (*n* = 3 HSV rings per group). Arrow indicates the internal elastic lamina. Int: neointima of HSV; Med: media tunica of HSV. (D) Representative images (left) and quantification (right) of PCNA immunofluorescence in HSV rings from the Ad‐NC and Ad‐FHL1 groups on Day 14 (*n* = 3 HSV rings per group). Arrows indicate PCNA(+) cells. Scale bar = 50 µm or 100 µm. L: lumen. ns: not significant, *p < .05, **p < .01, ***p < .001.

To elucidate the potential role of FHL1 in the pathogenesis of NIH in humans, its effects were assessed using an ex vivo static HSV ring culture model. Among the four tested concentrations, adenoviral infection efficiency exceeded 80% at 7$\;\times 10^{10}$ PFU/mL (Figure 9A). FHL1 overexpression was confirmed by treating HSV rings with Ad‐FHL1 or Ad‐NC at a concentration of 7$\times 10^{10}$ PFU/mL for 12 h, followed by a 14‐day culture. Immunofluorescence analysis showed a significant increase in FHL1 staining in the Ad‐FHL1 group compared to the Ad‐NC group (*p* < .001) (Figure 9B), indicating successful upregulation of FHL1 via adenoviral delivery in HSV rings. Therapeutic overexpression of FHL1 effectively prevented the development of NIH in HSV rings after 14 days, as compared to the empty virus treatment (*p* < .01) (Figure 9C). Furthermore, pre‐treatment with Ad‐FHL1 for 12 h led to a significant reduction in cell proliferation (*p* < .01) (Figure 9D). These results strongly suggest that FHL1 plays a critical role in human vein graft failure and highlight its regulatory function in the molecular mechanisms driving NIH in humans.

## 4 DISCUSSION

Omics‐based analyses of vein grafts play a vital role in unravelling occlusion mechanisms and identifying potential therapeutic targets. Tang et al. employed bulk RNA‐seq to investigate key molecules involved in preventing NIH but identified relatively nonspecific targets. Their immunohistochemical results indicated widespread expression of MFN2 across all graft regions.^31^ In contrast, ST technology offers a superior analysis of spatial heterogeneity within grafts, allowing for the direct identification of highly selective target molecules specifically within the neointimal region.^26^ In this study, a supervised clustering approach was utilised to construct a transcriptome atlas corresponding to three distinct cellular layers, incorporating spatial information more comprehensively.^32^, ^33^ Currently, no gene has been exclusively enriched in the neointima. This research leveraged the unique strengths of ST technology to, for the first time, identify marker genes specific to the neointima, which is essential for advancing targeted NIH therapies.

PCA and transcriptome proximity analysis revealed transcriptomic differences, while tissue staining suggested that neointimal cells likely originated from medial layer migration. Consequently, a comparison of DEGs between the neointima and media was performed to pinpoint genes exclusive to the neointimal region. Morphological assessments, along with GO and KEGG analyses, indicated that NIH may be linked to muscle development, actin cytoskeleton regulation, and focal adhesion. Among the 29 DEGs verified via immunohistochemistry, only Fhl1 was specifically enriched in the neointima. Co‐localisation fluorescence experiments confirmed that Fhl1 is expressed in hybrid phenotype neointimal cells. Additionally, in multiple replications, Fhl1 was consistently expressed exclusively in the neointima throughout all NIH stages, highlighting its strong reproducibility and stability. This study is the first to report the spatial expression distribution of Fhl1 in vein grafts. To date, Fhl1 exhibits the highest enrichment in the neointima during NIH phases compared to previously identified molecules, positioning it as a key biomarker and potentially a critical factor in NIH development.^31^, ^34^

Fhl1 was initially identified in skeletal muscle^35^, ^36^ and is primarily localised in the cytoplasm, where it participates in processes such as focal adhesion, sarcomere assembly, and cytoskeleton remodelling.^37^, ^38^ Its role in inflammation has been well‐documented. Colleen D'Arcy et al. observed that Fhl1 overexpression in mice reduced muscle inflammation, primarily by decreasing macrophage infiltration.^39^ Similarly, Ding et al. demonstrated that Fhl1 KO in mice significantly activated NLRP3 and other related inflammatory factors, intensifying muscle pyroptosis and apoptosis.^40^ In the context of atherosclerosis, it is known that VCAM‐1 promotes monocyte adhesion and activation into macrophages, thereby contributing to lesion progression.^41^, ^42^ Correspondingly, this study found that Fhl1 deletion led to a marked increase in neointimal area, primarily through the activation of the PI3K pathway, which enhanced VCAM‐1 expression. This upregulation, in turn, exacerbated macrophage infiltration and inflammation. Moreover, Fhl1 deficiency resulted in significantly heightened graft proliferative activity. Until now, the role of Fhl1 in vascular diseases has only been described in pulmonary hypertension.^43^ This study is the first to reveal that Fhl1 plays a critical role in inhibiting inflammation and proliferation, thus preventing NIH during vein graft remodelling.

To further explore Fhl1's clinical relevance, its molecular function was investigated in human veins. Given the limited availability of occluded vein grafts from patients undergoing coronary artery bypass grafting, an ex vivo culture model of human saphenous veins was established to simulate in vivo conditions.^44^, ^45^ In line with the rat model findings, adenovirus‐mediated overexpression of FHL1 effectively inhibited NIH in human saphenous veins. Thus, FHL1 appears to regulate NIH in humans and presents itself as a promising therapeutic target for preventing vein graft failure.

Most current research on vein graft remodelling primarily emphasises the role of SMCs in driving NIH.^13^, ^14^, ^15^, ^16^ However, this perspective overlooks the damage of multiple cellular components and the replacement of repair cells. Additionally, many studies have only observed vein grafts at limited time points.^19^, ^20^ In contrast, this study conducted dynamic observations across 11 time points, leading to several novel insights. First, nearly all SMCs in grafted veins undergo apoptosis, suggesting that, based on cell numbers, SMCs are unlikely to be the predominant source of NIH. Some earlier studies also noted the structural disappearance of medial SMCs.^46^, ^47^, ^48^, ^49^ Second, while previous research has often overlooked the changes at the graft boundaries,^18^ this study observed dynamic alterations in vessel borders and repair cells. *α*‐SMA(+) repair cells appeared within the cellulose layer outside the grafted veins, contributing to medial reconstruction. Subsequently, the original cellulose layer formed the adventitial layer, creating a new boundary. Some of these repair cells migrated through the ruptured IEL via amoeboid movement to form the neointima (Figure S15). When Fhl1 was completely knocked out in vivo, the distinction between the media and neointima spatial structures was lost, leading to increased NIH. This suggests that Fhl1 may be a spatially specific molecule critical for medial repair cells. Third, PCA and transcriptome proximity analyses revealed that by day 19, the grafts had diverged significantly from control veins, indicating fundamental changes in cellular gene composition. Based on these morphological findings, it can be inferred that newly generated α‐SMA(+) cells, rather than SMCs, from outside the graft may be the key cell population responsible for NIH.

A recent study demonstrated that after the transplantation of an artificial vascular graft into rats, the vascular wall exhibited normal vascular cell components.^50^ This is comparable to vein grafts, which serve as a fibrous scaffold for vascular remodelling. The repair cells primarily originate from living tissue surrounding the vessel rather than from the graft itself. Therefore, it can be speculated that interventions targeting the grafted vein may be less effective than those aimed at the surrounding tissues. This also provides a partial explanation for the failure of the E2F study to demonstrate a preventive effect in human trials.^51^, ^52^

Limitations: The evidence for cell migration in this study remains somewhat indirect. While cell labelling and tracing techniques can directly demonstrate migration, histological analysis across multiple time points with various stains provided robust evidence of migratory behaviour in the tissue samples. Due to the limited availability of graft tissues and cells, performing single‐cell RNA‐seq and ST across multiple time points was challenging. Consequently, accurately classifying neointimal cell types and comprehensively analysing the origin of neointimal cells proved difficult. Despite the inherent limitations of the *ex vivo* static HSV culture model—such as its inability to replicate haemodynamic conditions and cellular movement—it offers a readily accessible method in preclinical research for partially simulating human conditions and facilitating genetic modifications. With further refinement of our work, the prospect of clinical trials will become increasingly feasible.

## 5 CONCLUSIONS

In conclusion, cells outside grafted veins exhibiting hybrid SMC and fibroblast‐like phenotypes contribute to NIH. ST technology offers a comprehensive map of gene expression within the neointima, with Fhl1 consistently and stably expressed specifically in this region. Fhl1 plays a critical role in inhibiting inflammation and proliferation, thereby preventing NIH, laying a solid theoretical foundation for the precise identification and targeted treatment of NIH.

## AUTHOR CONTRIBUTIONS

MYL, ZKW designed the project; CQW, HLL, ZMZ, BHJ, ZYY performed the molecular analysis and experiments; JL, SQH, ZCF prepared figures; KNF, JTC analysed data; MYL, ZKW, CQW edited and revised the manuscript.

## CONFLICT OF INTEREST STATEMENT

The authors declare no competing interests.

## ETHICS STATEMENT

All animal experiments were performed with the approval of the Institutional Animal Care and Use Committee and conforms to the Guide for the Care and Use of Laboratory Animals of Sun Yat‐Sen University (Approval No. SYSU‐IACUC‐2020‐000074). All procedures conformed to the ARRIVE guidelines. Human saphenous vein experiments conformed to the BRISQ reporting guidelines and were approved by IEC for Clinical Research and Animal Trials of the First Affiliated Hospital of Sun Yat‐Sen University (Approval number: [2023]826).

## Supporting information



Supporting Information

## Data Availability

All data, code and reagents that support the findings of our study are available from the corresponding authors without restriction, and they take responsibility for its integrity and analysis. Further information and requests for resources should be directed to and will be fulfilled by the lead contact, Zhongkai Wu. The raw data have been deposited in the SRA database of NCBI BioSample Submission site under the accession number: PRJNA1166709 that are publicly accessible at https://www.ncbi.nlm.nih.gov/sra/PRJNA1166709.
