# Supplementary material for: Fhl1, a new spatially specific protein, regulates vein graft neointimal hyperplasia
Source: Clin Transl Med. 2024 Dec 5;14(12):e70115. doi: 10.1002/ctm2.70115 (PMC11621235; doi:10.1002/ctm2.70115)
Supplement: Supplementary file 1 — Supporting Information [file CTM2-14-e70115-s001.docx]

**Fhl1, a new spatially specific protein, regulates vein graft neointimal hyperplasia**

**Supplemental Figures, Tables and Methods**

**
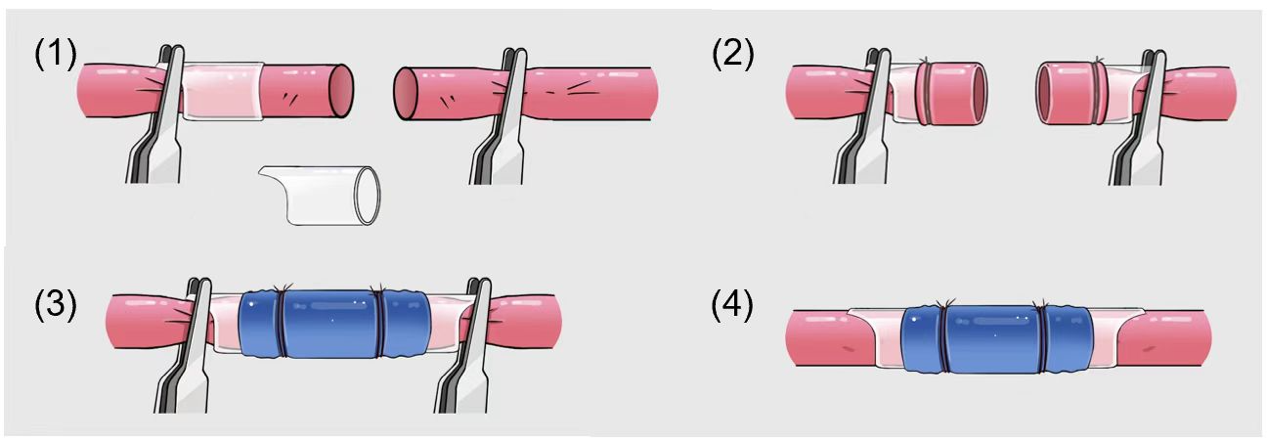
**

**
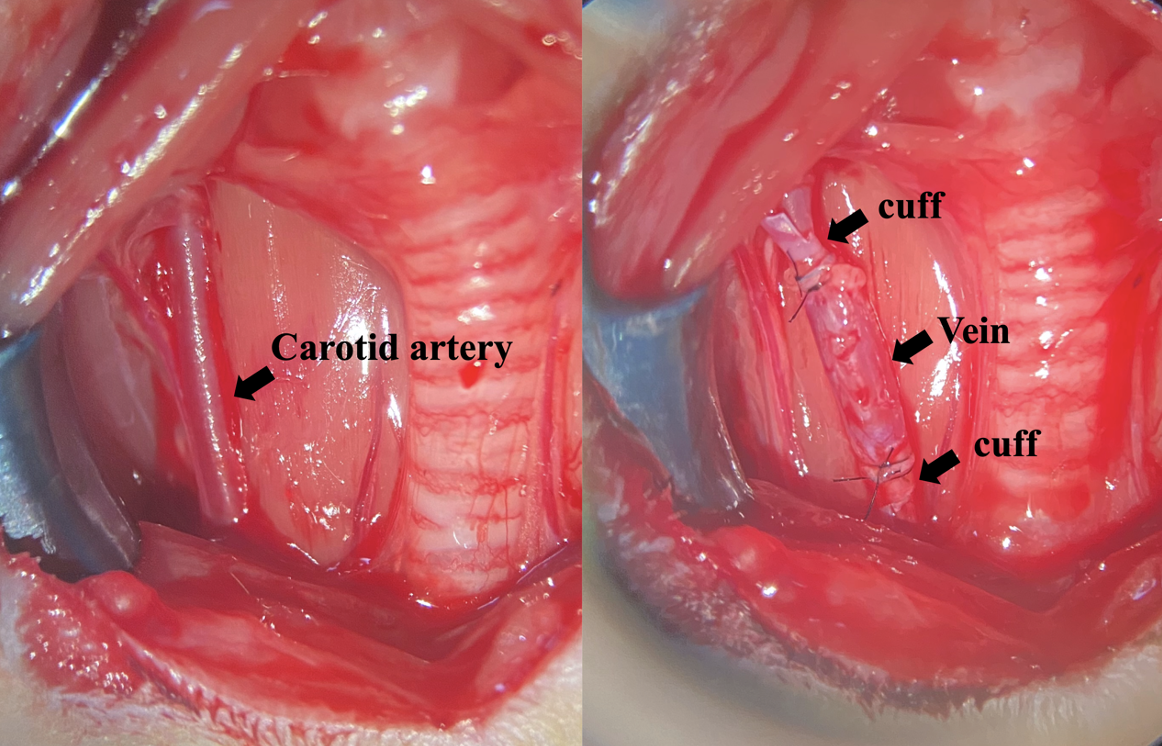
**

**Supplementary Figure 1.**

Schematic of the cuff technique of the arterialized vein graft model in rats (top panel). Surgical pictures of the external jugular vein grafted into the carotid artery (bottom panel).


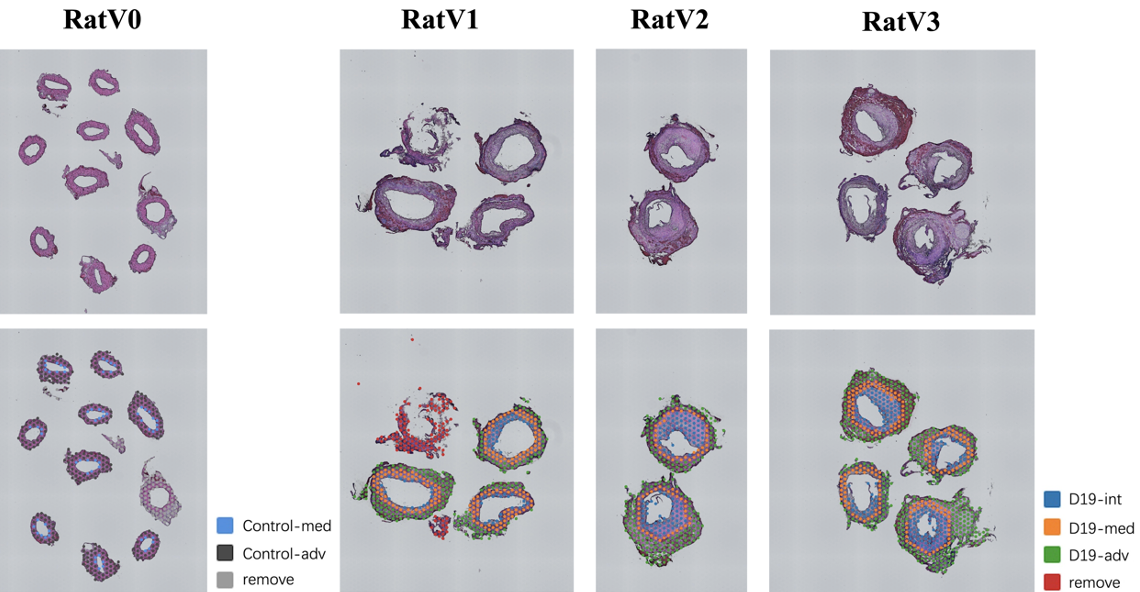


**Supplementary Figure 2.**

Ten different control veins were cryosectioned and assembled into one barcoded capture area (RatV0). Ten different Day 19 graft veins were sectioned and assembled into three barcoded capture areas (RatV1, RatV2, and RatV3). ST spots derived from unbiased clustering were overlaid upon each sample. Spots that did not cover intact vascular tissue were removed (n=1 from control veins and n=1 from Day 19 graft veins). Ctrl-med: media tunica of control external jugular vein; Ctrl-adv: adventitia of control external jugular vein; D19-int: neointima of Day 19th vein graft; D19-med: media tunica of Day 19th vein graft; D19-adv: adventitia of Day 19th vein graft.


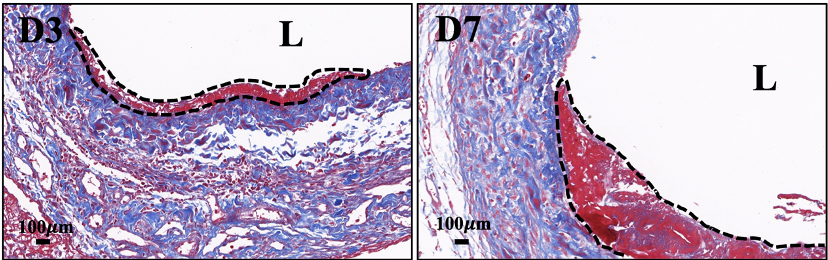


**Supplementary Figure 3.**

Fibrin formation in the lumen of the graft. Masson staining was performed 3 and 7 days after operation (n = 3). The dotted line indicates the fibrin in the lumen of the graft. L, lumen. Scale bar=100 *μ*m.

**
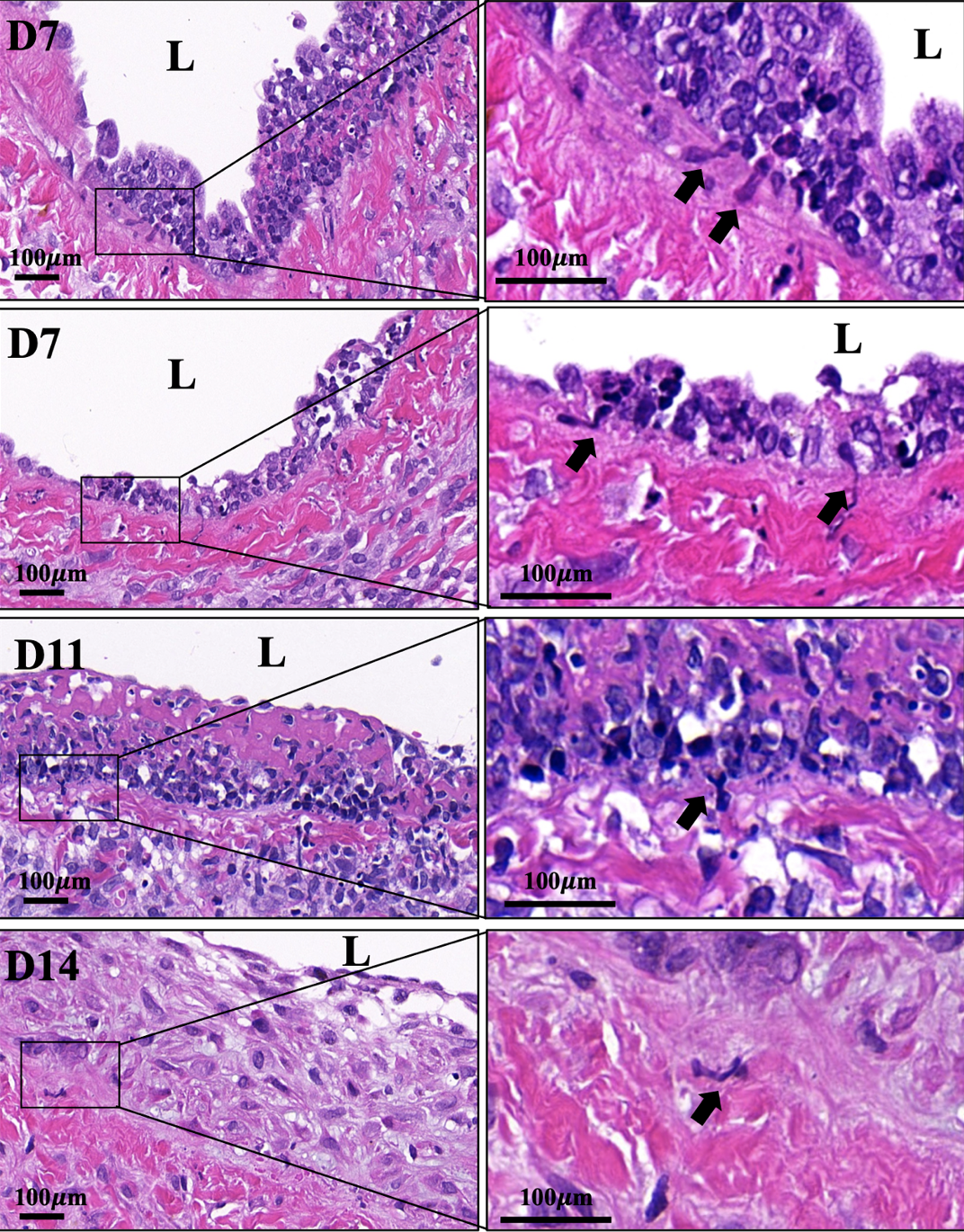
**

**Supplementary Figure 4.**

H&E staining revealed the evidence of cell migrating from media to neointima at 7, 11 and 14 days after operation. The black arrows indicate that the cells infiltrated across the disrupted IEL following by alteration of nuclear morphology. L, lumen. Scale bar=100 *μ*m.

**
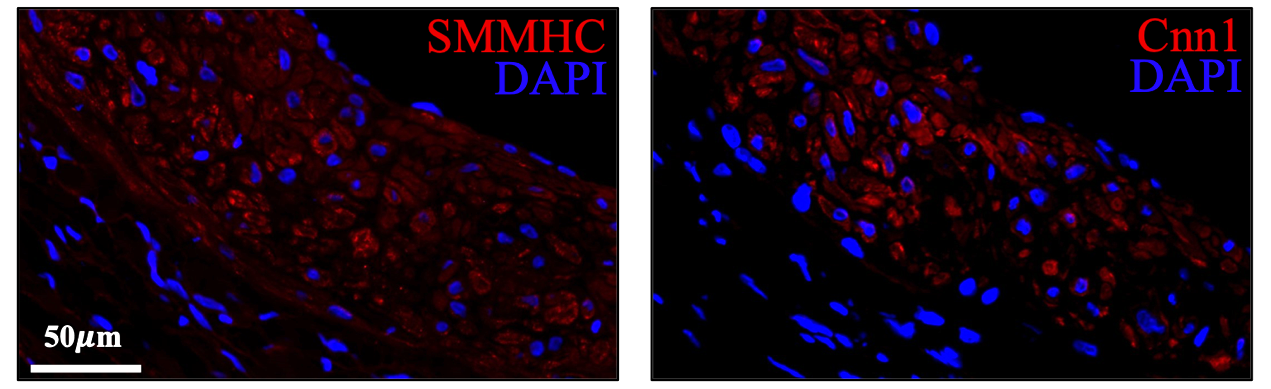
**

**Supplementary Figure 5.**

Late-stage(28d) neointimal cells exhibit the re-expression of SMMHC and Cnn1 (n = 3 rats per group). Representative IF images of DAPI (blue), SMMHC (red), or Cnn1 (red) staining in the neointima. Scale bar=50 *μ*m.

**
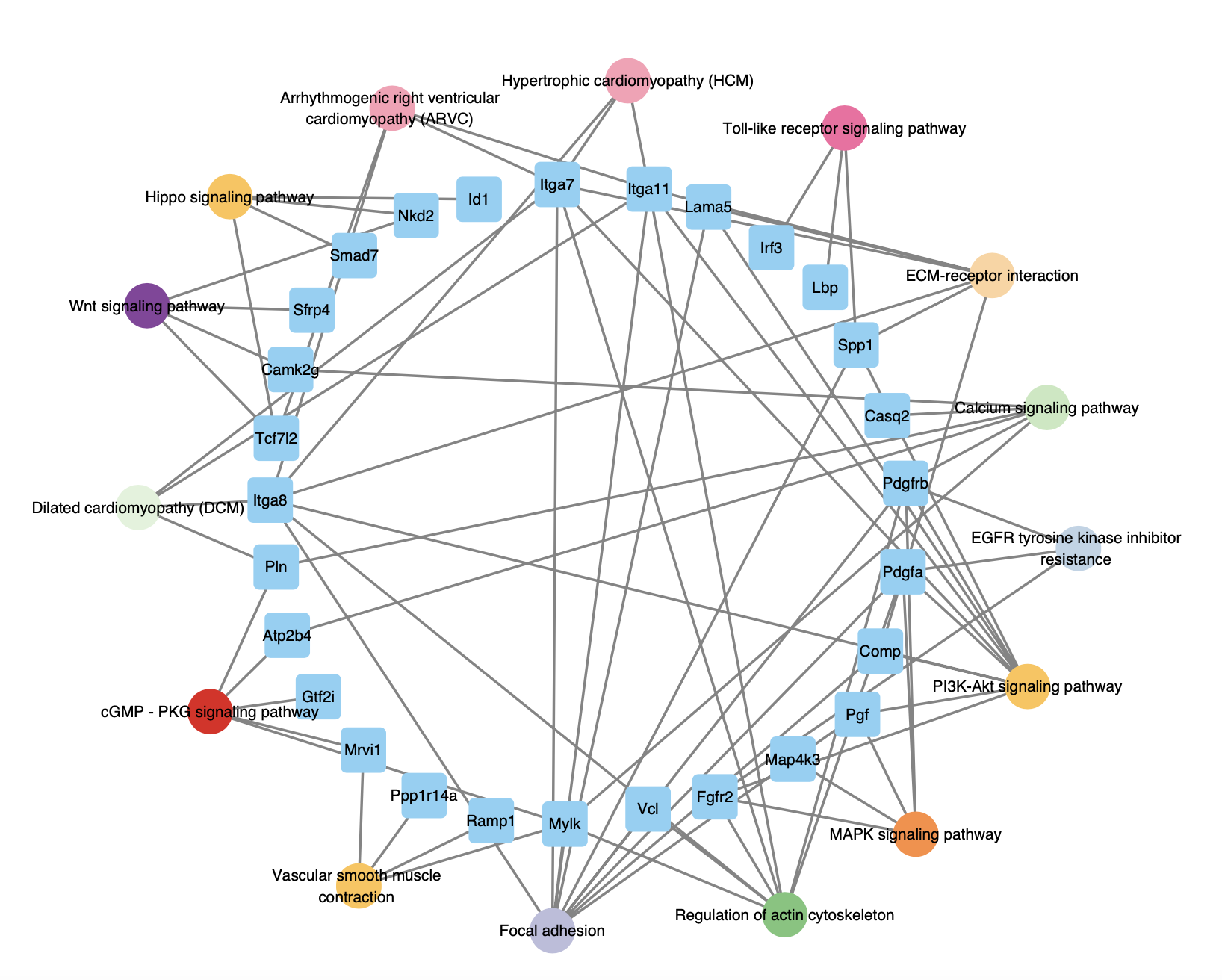
**

**Supplementary Figure 6.**

The networks of KEGG enriched pathways and significantly differentially expressed genes.

**
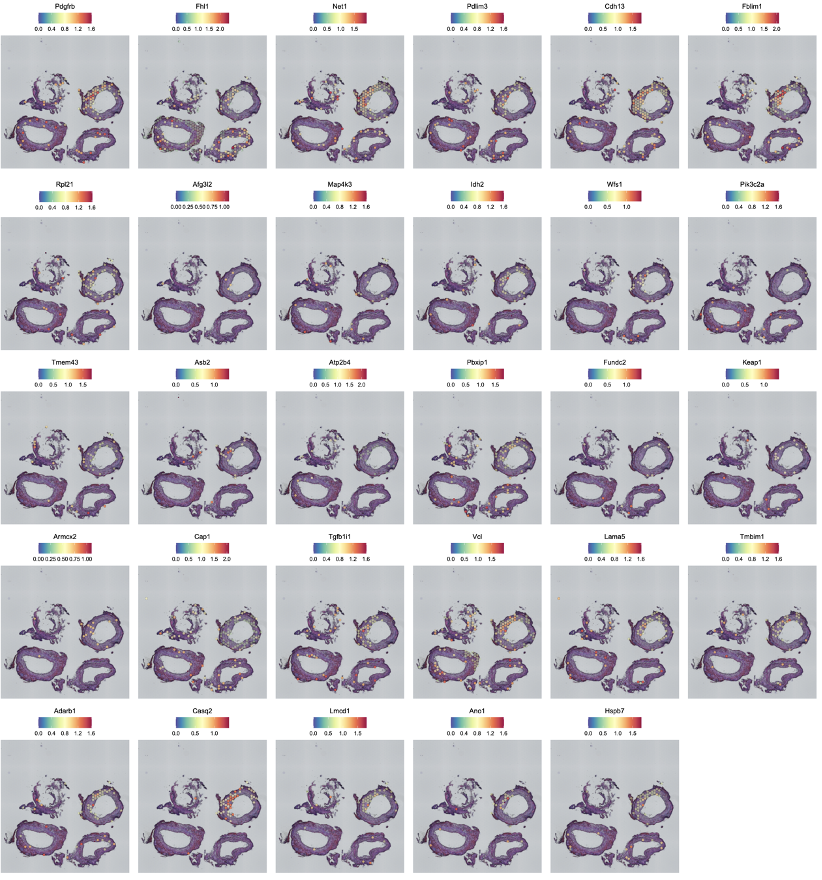
**

**Supplementary Figure 7.**

The 29 related upregulated DEGs were selected. These genes were interpolated across the tissue structure to visualize major spatial patterns within the day 19 graft veins. This figure represent RatV1 (n = 3).

**
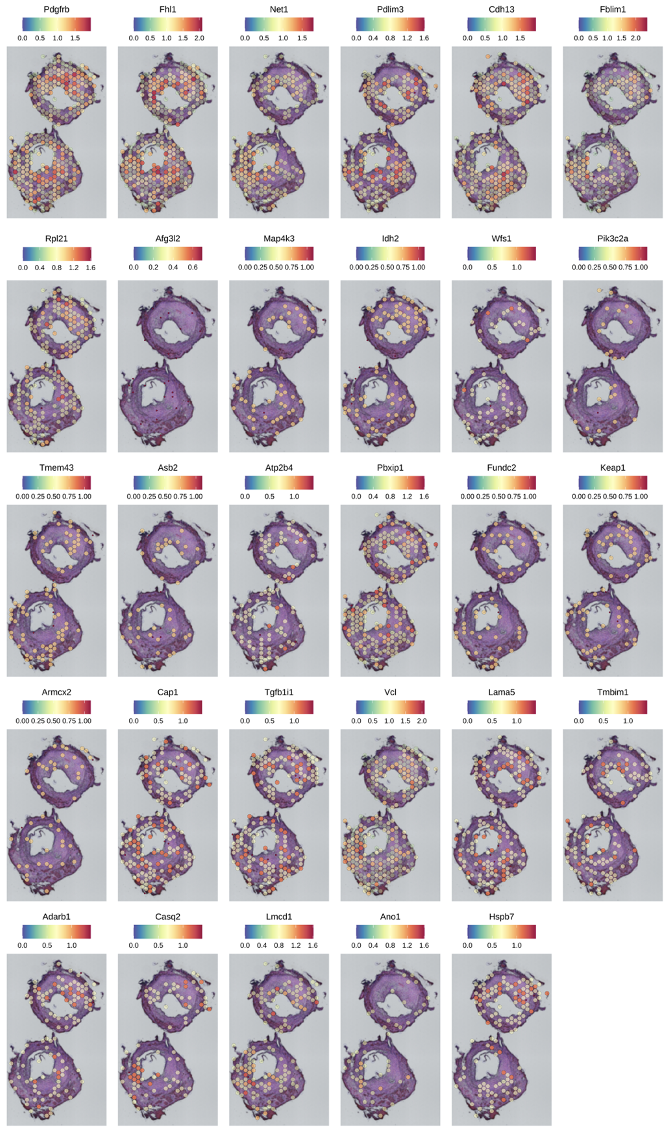
**

**Supplementary Figure 8.**

The 29 related upregulated DEGs were selected. These genes were interpolated across the tissue structure to visualize major spatial patterns within the day 19 graft veins. This figure represent RatV2 (n = 2).

**
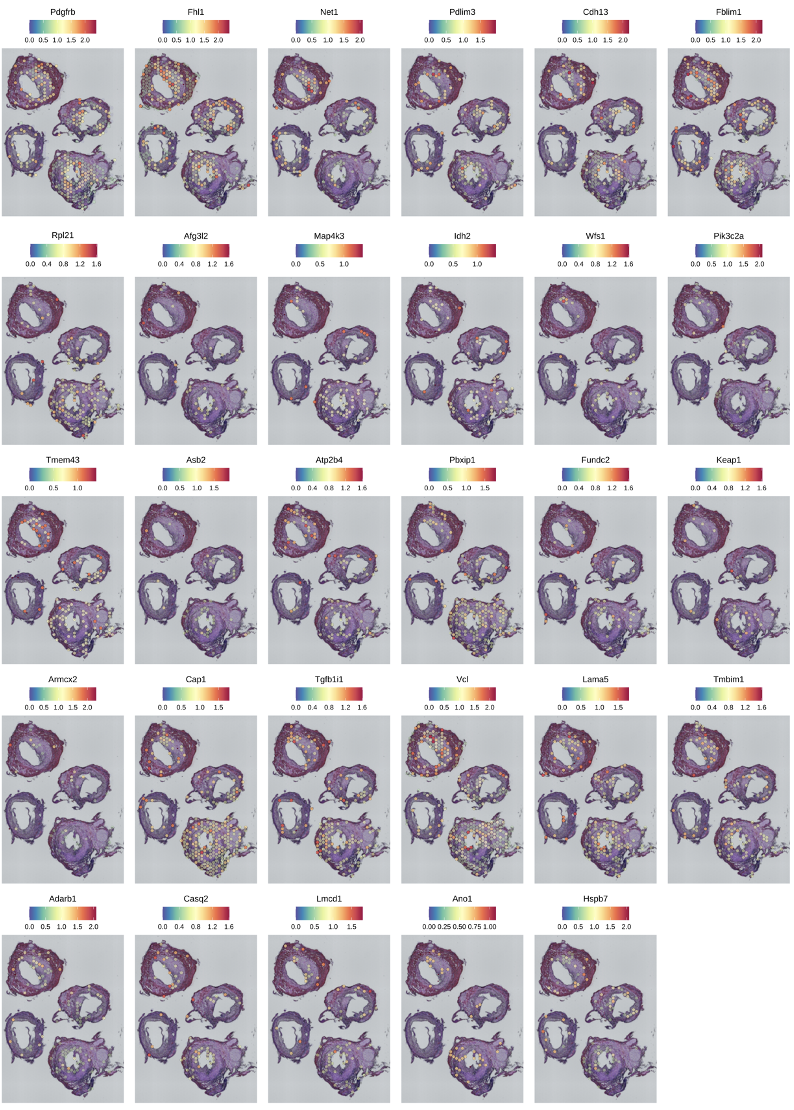
**

**Supplementary Figure 9.**

The 29 related upregulated DEGs were selected. These genes were interpolated across the tissue structure to visualize major spatial patterns within the day 19 graft veins. This figure represent RatV3 (n = 4).

**
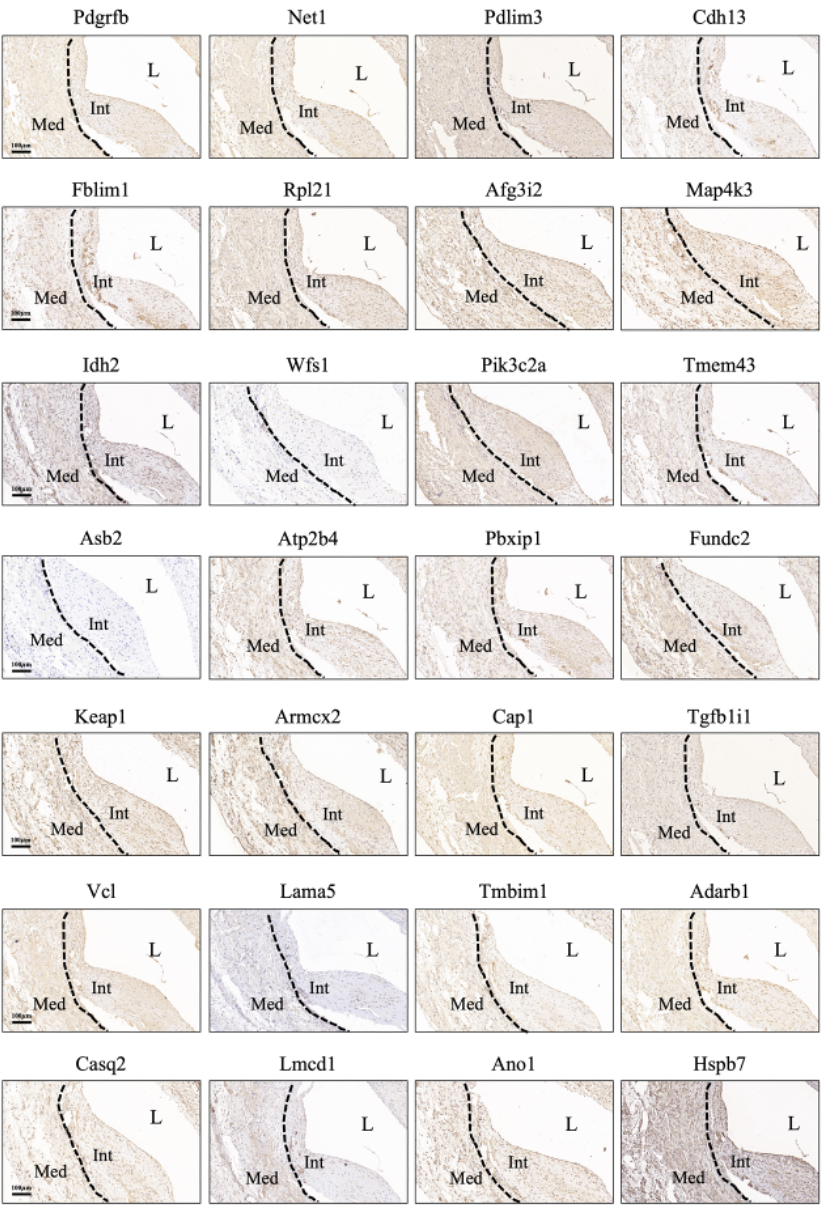
**

**Supplementary Figure 10.**

The 29 DEGs were verified by IHC staining in serial sections of grafts at 19 days. Int: neointima of vein graft; Med: media tunica of vein graft; Dotted line, internal elastic lamina. L, lumen. Scale bar=100 *μ*m.

**
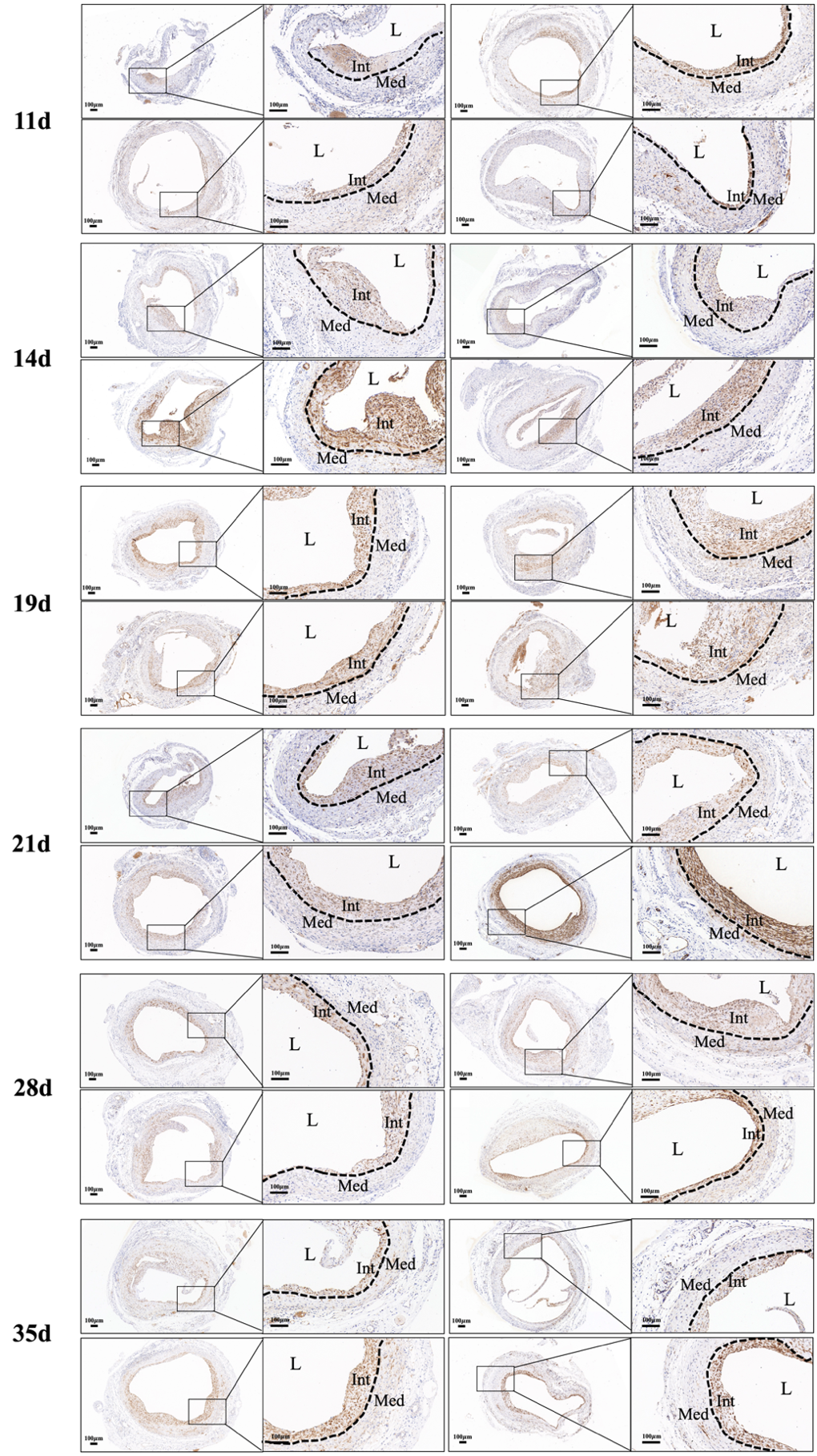
**

**Supplementary Figure 11.**

Remaining biologically independent samples from Fhl1 IHC staining (n = 4 rats per group). Int: neointima of vein graft; Med: media tunica of vein graft; Dotted line, internal elastic lamina. L, lumen. Scale bar=100 *μ*m.


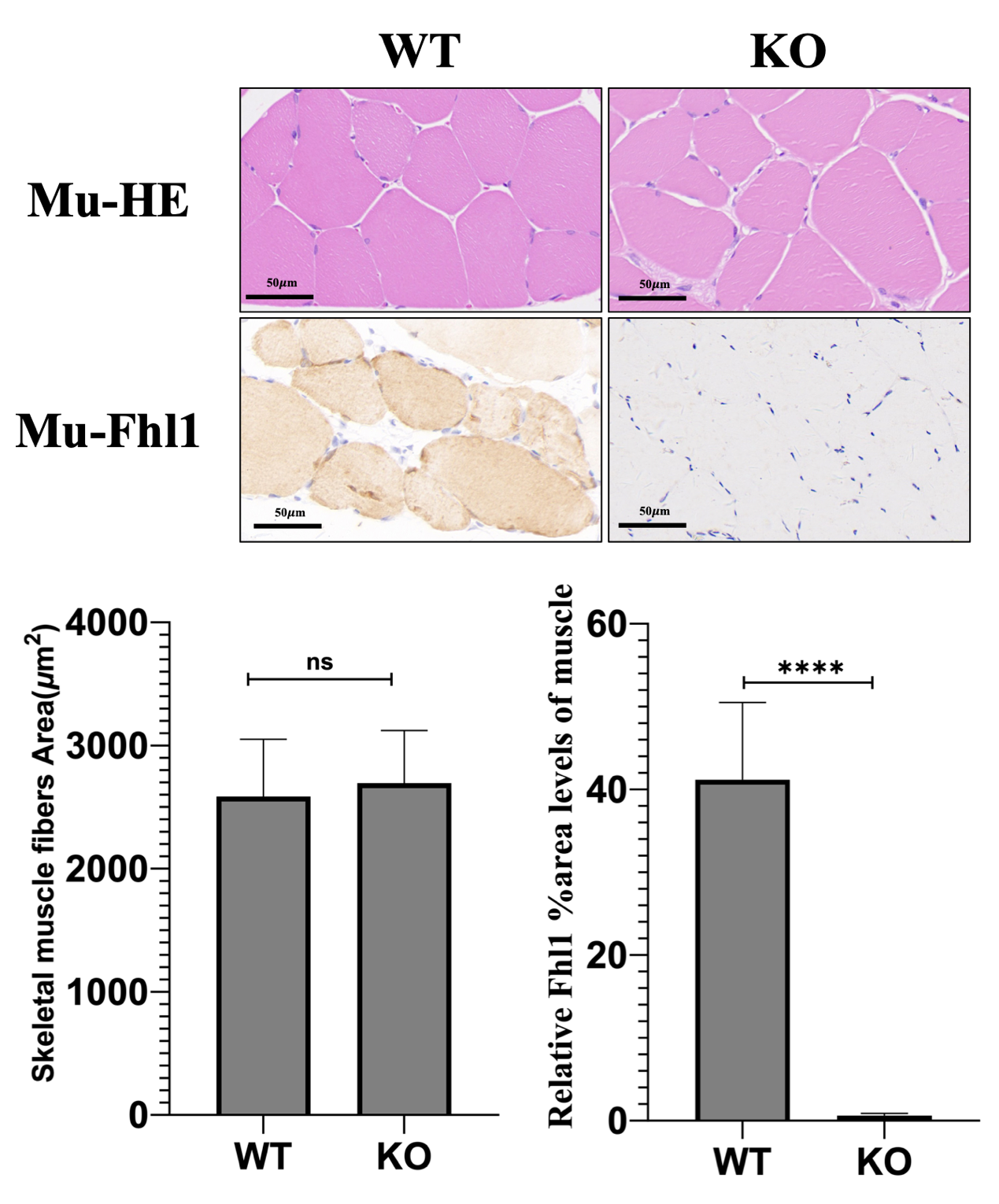


**Supplementary Figure 12.**

H&E stained and detection of Fhl1 expression by IHC of skeleton muscle from WT and Fhl1-KO rats (n=5 rats per group) (top panel). Quantification of muscle fibers area and the Fhl1 average regional staining intensity of skeleton muscle (bottom panel). Mu (muscle). *P* values correspond to two-tailed unpaired *t* test.


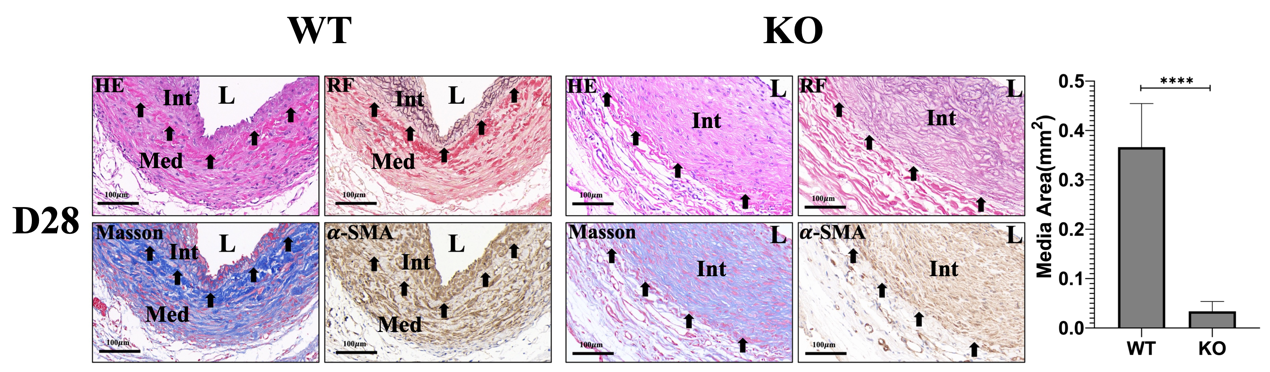


**Supplementary Figure 13.**

HE, Masson, RF, and *α*-SMA IHC staining of vein grafts (left) and quantification of the media area (right) of WT and Fhl1-KO rats at day 28 after grafted (n=8 rats per group). Arrow, internal elastic lamina. L: lumen. Int: neointima of graft; Med: media tunica of graft. Scale bar=100 μm.


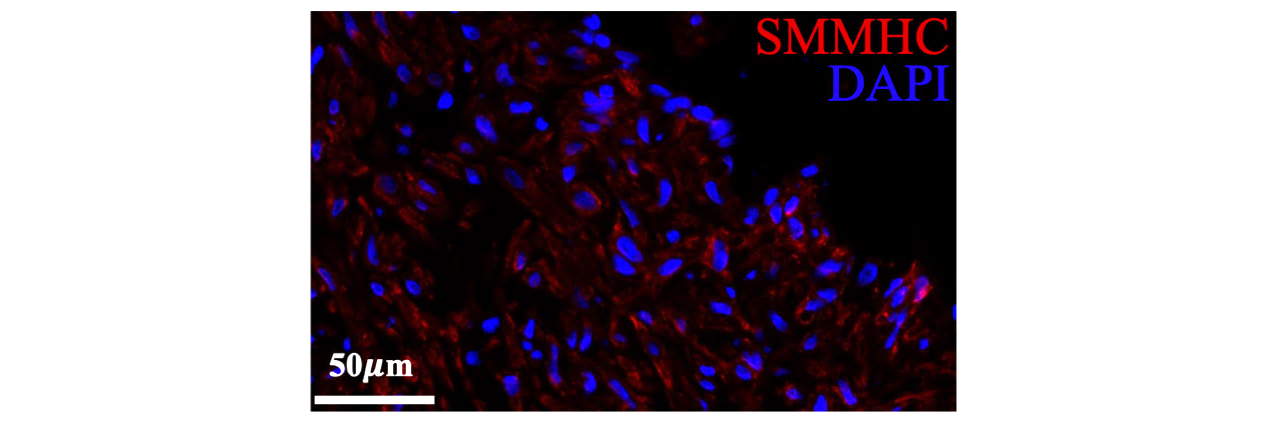


**Supplementary Figure 14.**

Late-stage neointimal cells from Fhl1 KO rats at 28 days exhibit the expression of SMMHC (n = 3). Representative IF images of DAPI (blue), SMMHC (red) staining in the neointima. Scale bar=50 *μ*m.


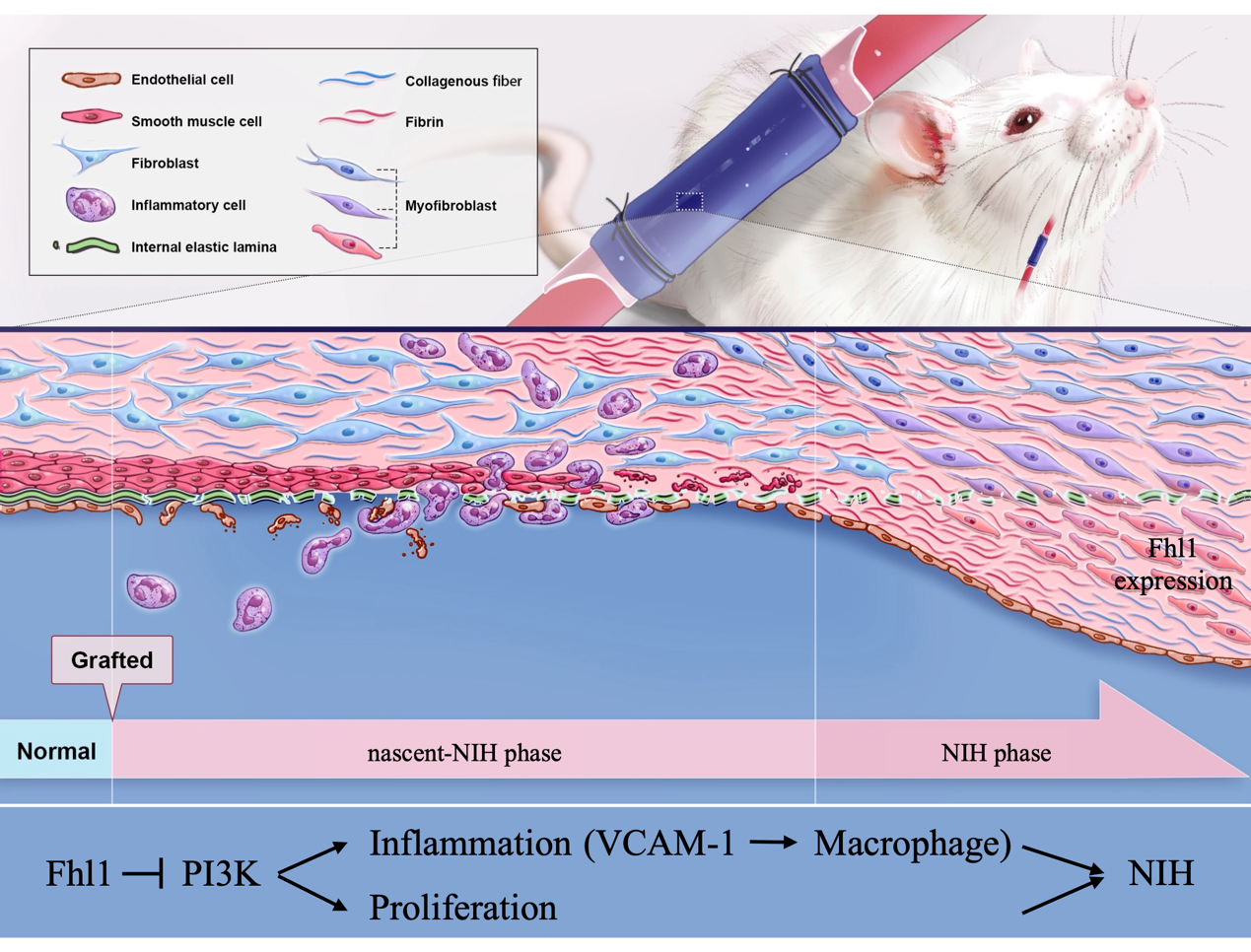


**Supplementary Figure 15.**

In the nascent-NIH phase, innate SMCs undergo apoptosis after grafting, and “new” medial cells are generated before NIH. By identifying morphological changes, these cells exhibited a hybrid phenotype between fibroblasts and SMCs, and a cell type transition to myofibroblasts is likely present. Hypothetically, these cells are regenerated inside the exudate, followed by alteration of phenotype and nuclear morphology, and migrate into media to replace the apoptotic cells and then contribute to NIH.

**Supplementary Table 1. Antibodies used for immunohistochemical staining**

| Antibody | Cat# | Manufacturer | Dilution |
| --- | --- | --- | --- |
| anti-α-SMA | ab124964 | Abcam, Cambridge, UK | 1:500 |
| anti-ERG | ab92513 | Abcam, Cambridge, UK | 1:300 |
| anti-MPO | ab208670 | Abcam, Cambridge, UK | 1:300 |
| anti-Fhl1 | ab255828 | Abcam, Cambridge, UK | 1:300 |
| anti-Pdgfrb | AF6133 | Affinity Biosciences, Cincinnati, USA | 1:100 |
| anti-Net1 | DF6345 | Affinity Biosciences, Cincinnati, USA | 1:100 |
| anti-Pdlim3 | DF12525 | Affinity Biosciences, Cincinnati, USA | 1:100 |
| anti-Cdh13 | AF5203 | Affinity Biosciences, Cincinnati, USA | 1:100 |
| anti-Fblim1 | DF13004 | Affinity Biosciences, Cincinnati, USA | 1:100 |
| anti-Rpl21 | 28470 | Signalway Antibody, Maryland, USA | 1:100 |
| anti-Afg3i2 | 29211 | Signalway Antibody, Maryland, USA | 1:100 |
| anti-Map4k3 | DF3342 | Affinity Biosciences, Cincinnati, USA | 1:100 |
| anti-Idh2 | 39188 | Signalway Antibody, Maryland, USA | 1:100 |
| anti-Wfs1 | ab259362 | Abcam, Cambridge, UK | 1:5000 |
| anti-Pik3c2a | orb374187 | Biorbyt Ltd, Cambridge, UK | 1:200 |
| anti-Tmem43 | ab190793 | Abcam, Cambridge, UK | 1:250 |
| anti-Asb2 | ta808134s | Origene, Rockvile, USA | 1:150 |
| anti-Atp2b4 | DF13326 | Affinity Biosciences, Cincinnati, USA | 1:100 |
| anti-Pbxip1 | DF12129 | Affinity Biosciences, Cincinnati, USA | 1:100 |
| anti-Fundc2 | orb450243 | Biorbyt Ltd, Cambridge, UK | 1:200 |
| anti-Keap1 | 32450 | Signalway Antibody, Maryland, USA | 1:200 |
| anti-Armcx2 | 40613 | Signalway Antibody, Maryland, USA | 1:300 |
| anti-Cap1 | DF13310 | Affinity Biosciences, Cincinnati, USA | 1:100 |
| anti-Tgfb1i1 | K108581P | Solarbio Science, Beijing, China | 1:100 |
| anti-Vcl | 32418 | Signalway Antibody, Maryland, USA | 1:100 |
| anti-Lama5 | ta322581 | Origene, Rockvile, USA | 1:50 |
| anti-Tmbim1 | 41391 | Signalway Antibody, Maryland, USA | 1:300 |
| anti-Adarb1 | DF3227 | Affinity Biosciences, Cincinnati, USA | 1:100 |
| anti-Casq2 | DF8008 | Affinity Biosciences, Cincinnati, USA | 1:200 |
| anti-Lmcd1 | ta503139s | Origene, Rockvile, USA | 1:150 |
| anti-Ano1 | DF7769 | Affinity Biosciences, Cincinnati, USA | 1:200 |
| anti-Hspb7 | FNab04060 | Fine Biotech, Wuhan, China | 1:50 |

**Supplementary Table 2. Antibodies used for immunofluorescence staining**

| Antibody | Cat# | Manufacturer | Dilution |
| --- | --- | --- | --- |
| anti-α-SMA | ab124964 | Abcam, Cambridge, UK | 1:250 |
| anti-PI3K | ab191606 | Abcam, Cambridge, UK | 1:200 |
| anti-Fhl1 | ab255828 | Abcam, Cambridge, UK | 1:50 |
| anti-Ki67 | GB121141 | Servicebio Technology, Wuhan, China | 1:200 |
| anti-Vimentin | GB12192 | Servicebio Technology, Wuhan, China | 1:400 |
| anti-SMMHC | GB11805 | Servicebio Technology, Wuhan, China | 1:250 |

**Supplementary Table 3. Overview of samples and reads quality, sequencing saturation, and effective barcode of sequencing data evaluation**

| Sample | Number of Reads | Valid Barcodes | Sequencing Saturation | Q30 Bases in Barcode | Q30 Bases in RNA Read | Q30 Bases in UMI |
| --- | --- | --- | --- | --- | --- | --- |
| RatV0 | 259574346 | 97.11% | 96.81% | 95.70% | 88.67% | 93.14% |
| RatV1 | 201207316 | 97.01% | 93.62% | 95.77% | 88.80% | 93.06% |
| RatV2 | 238982754 | 97.13% | 94.62% | 95.97% | 87.84% | 93.54% |
| RatV3 | 230588975 | 97.02% | 94.45% | 95.87% | 90.13% | 93.44% |

**Supplementary Table 4.** **Overview of samples and the number of genes, the number of valid reads and other data evaluation**

| Sample | Number of Spots Under Tissue | Fraction Reads in Spots Under Tissue | Mean Reads per Spot | Median Genes per Spot | Total Genes Detected |
| --- | --- | --- | --- | --- | --- |
| RatV0 | 440 | 54.01% | 589941.7 | 1056.5 | 12576 |
| RatV1 | 573 | 47.03% | 351147.15 | 384 | 13155 |
| RatV2 | 380 | 58.80% | 628901.98 | 2654.5 | 13444 |
| RatV3 | 744 | 68.23% | 309931.42 | 870.5 | 14174 |

| Median UMI Counts per Spot | Reads Mapped Confidently to Genome | Reads Mapped Confidently to Intergenic Regions | Reads Mapped Confidently to Intronic Regions | Reads Mapped Confidently to Exonic Regions | Reads Mapped Confidently to Transcriptome |
| --- | --- | --- | --- | --- | --- |
| 2657.5 | 77.10% | 11.93% | 2.70% | 62.47% | 57.85% |
| 704 | 68.21% | 15.03% | 2.31% | 50.88% | 47.57% |
| 8693.5 | 72.55% | 12.47% | 2.81% | 57.27% | 53.56% |
| 1686 | 80.01% | 13.19% | 3.33% | 63.49% | 59.34% |

**Supplementary Table 5.**

All of the upregulated DEGs were outputted in the ascending order of adjust P. See relevant excel

**Supplementary method 1.**

The sequence information of FHL1 inserted into the Ad was as follows:

atggcggagaagtttgactgccactactgcagggatcccttgcaggggaagaagtatgtgcaaaaggatggccaccactgctgcctgaaatgctttgacaagttctgtgccaacacctgtgtggaatgccgcaagcccatcggtgcggactccaaggaggtgcactataagaaccgcttctggcatgacacctgcttccgctgtgccaagtgccttcaccccttggccaatgagacctttgtggccaaggacaacaagatcctgtgcaacaagtgcaccactcgggaggactcccccaagtgcaaggggtgcttcaaggccattgtggcaggagatcaaaacgtggagtacaaggggaccgtctggcacaaagactgcttcacctgtagtaactgcaagcaagtcatcgggactggaagcttcttccctaaaggggaggacttctactgcgtgacttgccatgagaccaagtttgccaagcattgcgtgaagtgcaacaaggccatcacatctggaggaatcacttaccaggatcagccctggcatgccgattgctttgtgtgtgttacctgctctaagaagctggctgggcagcgtttcaccgctgtggaggaccagtattactgcgtggattgctacaagaactttgtggccaagaagtgtgctggatgcaagaaccccatcactgggaaaaggactgtgtcaagagtgagccacccagtctctaaagctaggaagcccccagtgtgccacgggaaacgcttgcctctcaccctgtttcccagcgccaacctccggggcaggcatccgggtggagagaggacttgtccctcgtgggtggtggttctttatagaaaaaatcgaagcttagcagctcctcgaggcccgggtttggtaaaggctccagtgtggtggcctatgaaggacaatcctggcacgactactgcttccactgcaaaaaatgctccgtga
